# Supplementary material for: Interdependence between EGFR and Phosphatases Spatially Established by Vesicular Dynamics Generates a Growth Factor Sensing and Responding Network
Source: Cell Syst. 2018 Sep 26;7(3):295–309.e11. doi: 10.1016/j.cels.2018.06.006 (PMC6167251; doi:10.1016/j.cels.2018.06.006)
Supplement: Document S1. Figures S1–S4 [file mmc1.pdf]

Cell Systems, Volume 7

Supplemental Information

Interdependence between EGFR and Phosphatases Spatially Established by Vesicular Dynamics Generates a Growth Factor Sensing and Responding Network

Angel Stanoev, Amit Mhamane, Klaus C. Schuermann, Hernán E. Grecco, Wayne Stallaert, Martin Baumdick, Yannick Brüggemann, Maitreyi S. Joshi, Pedro Roda-Navarro, Sven Fengler, Rabea Stockert, Lisaweta Roßmannek, Jutta Luig, Aneta Koseska, and Philippe I.H. Bastiaens

Supplemental Information

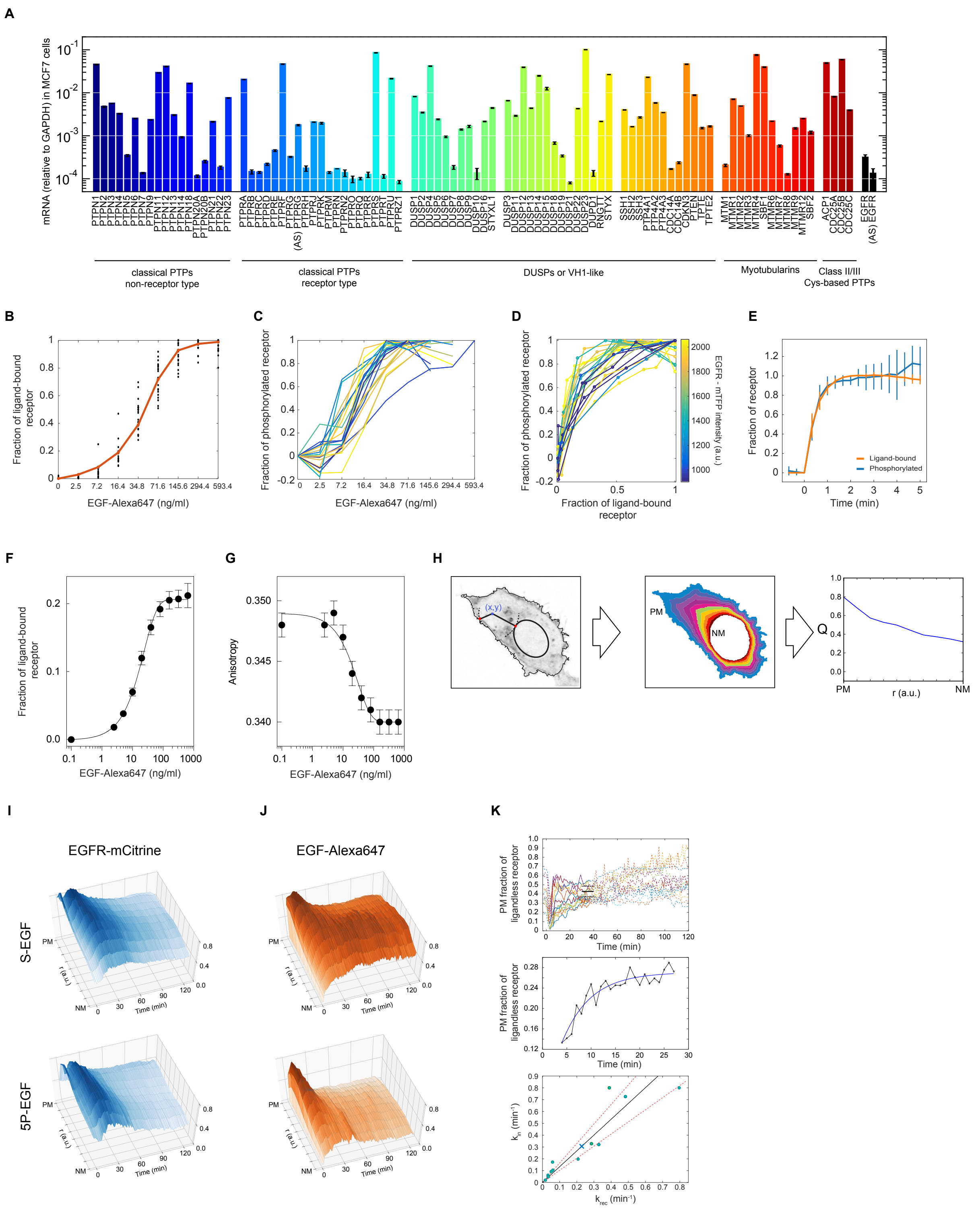

Figure S1. EGFR phosphorylation and trafficking dynamics, related to Figure 1. (A) PTP_X and EGFR mRNA expression relative to GAPDH mRNA in MCF7 cells (means±SD) measured by microarray analysis. AS: anti-sense. **(B)** Estimating fraction of EGFR-mTFP bound to EGF-Alexa647 in live cells: EGF-Alexa647/EGFR-mTFP quantified in single cells (data points) upon increasing EGF-Alexa647 doses was fitted (line) with a receptor binding

kinetics model (STAR Methods). **(C)** Single cell profiles of the fraction of phosphorylated EGFR-mTFP, quantified by PTB-mCherry translocation to the plasma membrane ($\frac{[PTB-mCherry_{PM}]/[PTB-mCherry]_T}{[EGFR-mTFP_{PM}]/[EGFR-mTFP]_T}$, STAR Methods) for increasing EGF-Alexa647 doses. **(D)** Single cell EGFR-mTFP phosphorylation response versus fraction of ligand bound receptors (mean±SD shown in Figure 1D) derived from (**B**) and (**C**). The estimated fractions of phosphorylated vs. liganded EGFR-mTFP are plotted and color-coded by the average EGFR-mTFP fluorescence intensity per cell. **(E)** Quantification of PTB-mCherry translocation kinetics to plasma membrane localized EGFR-mTFP. MCF7 cells were stimulated with a saturating EGF-Alexa647 dose (320ng/ml) and successive images were acquired every 20s (n=10, means±SD). Translocated plasma membrane fraction of PTB-mCherry ($\frac{[PTB-mCherry_{PM}]/[PTB-mCherry]_T}{[EGFR-mTFP_{PM}]/[EGFR-mmTFP]_T}$) converged to a steady state level in ~1.5min, which was within the time frame of successive EGF-Alexa647 dose administration (Figure 1C-D). **(F)** Estimation of the fraction of EGFR-QG-mCitrine bound to EGF-Alexa647 in live cells (n=30, N=3, means±SEM) from fluorescence anisotropy microscopy is equivalent to the corresponding estimation from confocal microscopy with EGFR-mTFP (Figure S1B). **(G)** Dependency of the EGFR-QG-mCitrine dimerization state on increasing EGF-Alexa647 doses determined by fluorescence anisotropy microscopy (n=30, N=3, means±SEM). **(H)** Dimensionality reduction from Cartesian (x, y) to normalized radial (r) distribution of quantity (Q) between the plasma (PM) and the nuclear (NM) membrane. **(I)** Average spatial-temporal maps (STMs) of EGFR-mCitrine intensity obtained from live cells stimulated with 200ng/ml S-EGF (n=16, N=3; top) and 5P-EGF (n=14, N=2; bottom). **(J)** Corresponding STMs of EGF-Alexa647 fluorescence. **(K)** Quantification of recycling dynamics of ligandless EGFR-mCitrine upon 200ng/ml 5P-EGF. Top: plasma membrane fraction of ligandless EGFR-mCitrine in single cells. Model-based estimation of the steady state level (95% confidence bounds; see STAR Methods) is shown with black line (inside red dashed lines). Middle: compartment-model-based fitting on 4-35min time interval for the cells shown in the bottom panel of Figure 1G (estimated rates: k_{in}=0.31min^{-1} (0.12, 0.50) 95% confidence bounds, k_{rec}=0.23min^{-1} (0.08, 0.38), STAR Methods). Bottom: Linear dependency between (k_{in}, k_{rec}) reflects that similar steady state plasma membrane fraction of ligandless EGFR are maintained in different cells by recycling (x: average (k_{in}, k_{rec}); STAR Methods). Linear fit (black line) with 95% confidence interval half-width (dashed red lines) is shown.

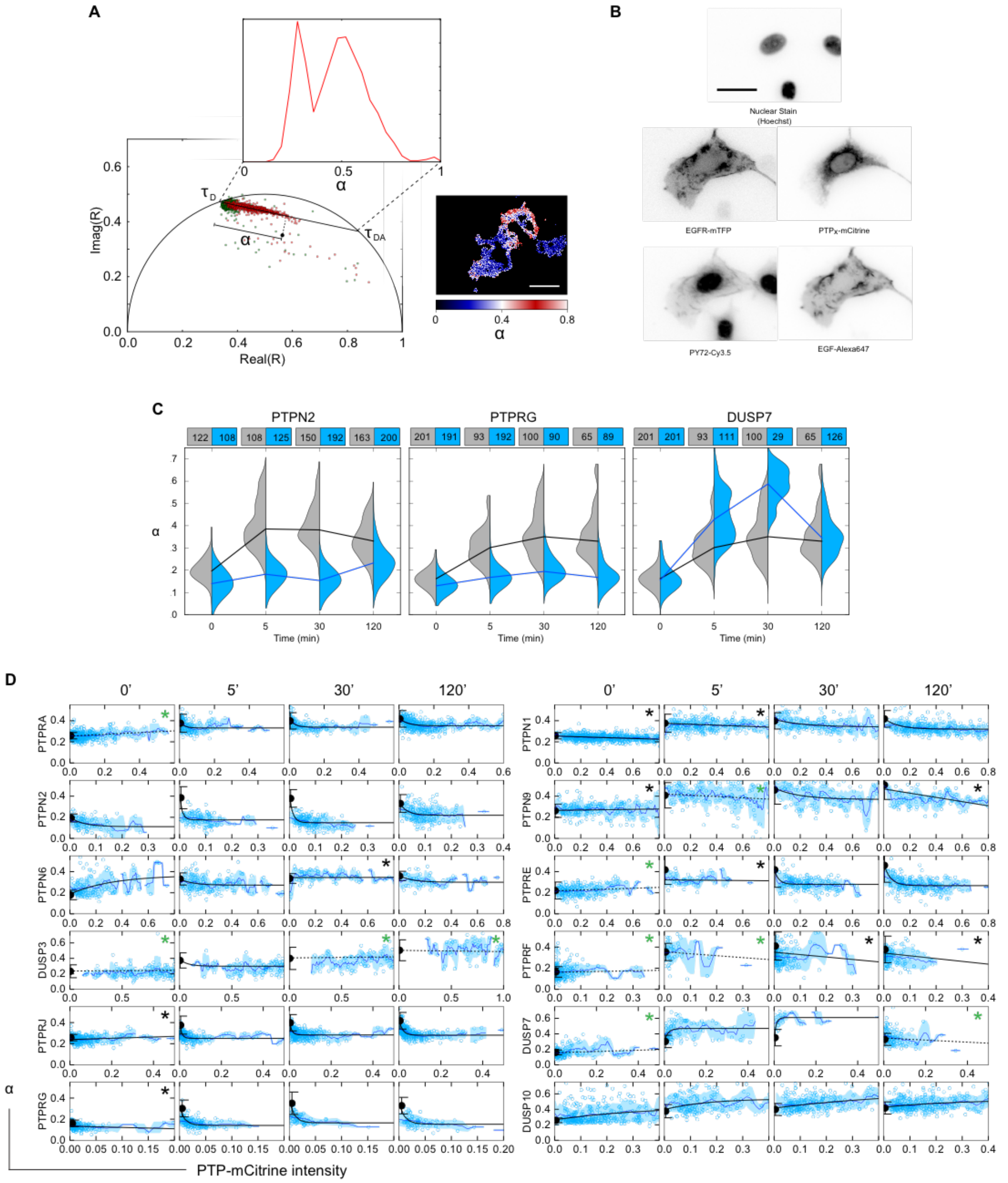

Figure S2. CA-FLIM and related quantities, Related to Figure 2. (A) Linear fit of the fluorescence emission Fourier coefficients (R) in the complex plane yielding the global EGFR-mTFP lifetimes in presence (τ_{DA}) and absence (τ_D) of FRET. Fraction of phosphorylated EGFR-mTFP bound by PY72-Cy3.5 (α) in each pixel was calculated from the projection onto the τ_D-τ_{DA} line segment. An exemplary α-histogram (inset) and a spatially resolved α-map (right) are shown. Scale bar: 20µm. **(B)** Representative images obtained in CA-FLIM screen: Hoechst (nuclear stain), EGFR-mTFP, PTP_X-mCitrine, PY72-Cy3.5 and EGF-Alexa647 fluorescence. Scale bar: 10µm. **(C)** Exemplary temporal EGFR-mTFP

phosphorylation profiles (grey, control) upon ectopic PTP_X-mCitrine expression (PTP_X=PTPN2, PTPRG, DUSP7; blue). The violin plots show the α distributions from single cells stimulated with 200ng/ml 5P-EGF (number of cells denoted on top of the plots, medians at different time points are connected by a black line). **(D)** α vs. PTP_X-mCitrine single cell fluorescence scatter plots. Black circle: mean α_{ctr}±SD; black lines: exponential fits (* - linear fit for weak dependence, green asterisk: distributions of α_{ctr} and α_{PTP} did not significantly differ); blue lines with error bounds: moving averages with standard deviations. The slope of the exponential (or linear) fit at the intercept is defined as the relative specific PTP_X-mCitrine activity in Figure 2C (middle).

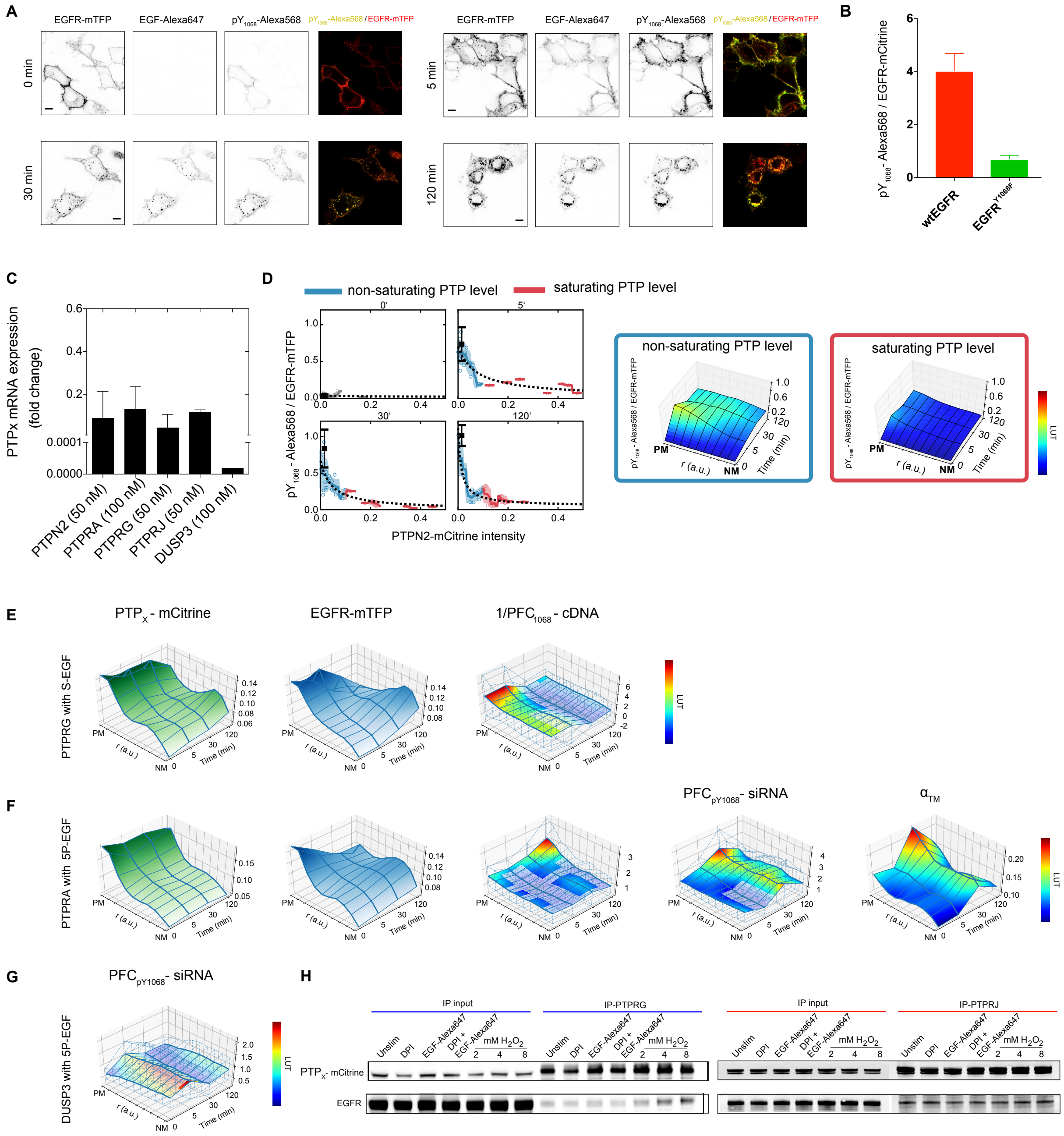

Figure S3. PTPs shape EGFR phosphorylation response, Related to Figure 3. (A) Representative images of EGFR-mTFP, EGF-Alexa647, anti-pY_{1068}-Alexa568 fluorescence and overlay of pY_{1068} (yellow) and EGFR-mTFP (red) prior to and after 5, 30 and 120min of 5P-EGF stimulation (200ng/ml) of MCF7 cells. Scale bar: 50µm. **(B)** Binding of pY_{1068}-Alexa568 antibody to pY_{1068} on EGFR-mCitrine and $EGFR^{Y1068F}$-mCitrine reflects its specificity for the corresponding tyrosine phosphorylation site (mean±SD, n~100, N=1). **(C)** mRNA expression fold change of PTPN2, PTPRG, PTPRJ, PTPRA and DUSP3 in MCF7 cells after 24h transfection with 50nM or 100nM respective siRNA. The values are relative to the corresponding mRNA levels of cells treated with 50nM non-targeting siRNA for 24hr (means±SEM, N=2, and for DUSP3 N=1). **(D)** Left: example of pY_{1068}-Alexa568/EGFR-

mTFP vs. PTPN2-mCitrine fluorescence intensity scatter plots used to determine the PTPN2-mCitrine fluorescence intensity threshold at which saturation of EGFR dephosphorylation occurs. Blue/red circles represent single cells below and above the PTPN2-mCitrine fluorescence intensity threshold, respectively; solid lines and shaded bounds: corresponding moving averages and standard deviation. The data was fitted with a function depicting the dependency of pY_{1068}/EGFR-mTFP on PTP_X-mCitrine intensity (steady state EGFRp assumption, STAR Methods). Right: spatial temporal maps (STMs) of pY_{1068}/EGFR-mTFP averaged from cells below (blue box) and above (red box) PTPN2-mCitrine fluorescence intensity threshold. **(E)** Effect of PTPRG-mCitrine expression (left) on STMs of EGFR-mTFP fluorescence (middle) and phosphorylation fold-change (1/PFC_{pY1068}-cDNA, right) reflecting the relative PTPRG-mCitrine reactivity towards pY_{1068} for cells stimulated with 200ng/ml S-EGF (n~30). **(F)** Columns 1-3: effect of PTPRA-mCitrine expression (Column 1) on STMs of EGFR-mTFP localization (Column 2) and phosphorylation fold-change (1/PFC_{pY1068}-cDNA, Column 3), which reflects the relative PTPRA-mCitrine reactivity towards pY_{1068} (n~60, N=3). Column 4: effect of siRNA-mediated PTPRA knock-down on EGFR-mTFP phosphorylation fold change (PFC_{pY1068}-siRNA, n~45, N=3). Column 5: STM of fraction of EGFR-mTFP interacting with PTPRA ^{C442S}-mCitrine trapping mutant (α_{TM}, n=15-30, N=2). **(G)** Effect of siRNA-mediated DUSP3 knock-down on EGFR-mTFP phosphorylation fold change (PFC_{pY1068}-siRNA, n~40, N=3). In (F-G), cells were stimulated with 200ng/ml 5P-EGF; transparent areas in (E-G): non-significant PFCs, p >0.05. LUT: look-up table. **(H)** Identifying and characterizing the interaction between EGFR and PTPRG/J-mCitrine by co-immunoprecipitation. Co-immunoprecipitated EGFR (second row) following PTPRG- (left) or PTPRJ-mCitrine (right) pull down from MCF7 cells prior to and after treatment with DPI (20min, 10μM), EGF-Alexa647 (10 min, 200ng/ml), DPI pretreatment (20min, 10μM) followed by EGF-Alexa647 (10 min, 200ng/ml) and H_2O_2 (10 min; 2, 4 or 8 mM) by western blotting. IP input: total expressed protein, IP-PTPRG/J: PTPRG/J-mCitrine immunoprecipitated by anti-GFP antibody.

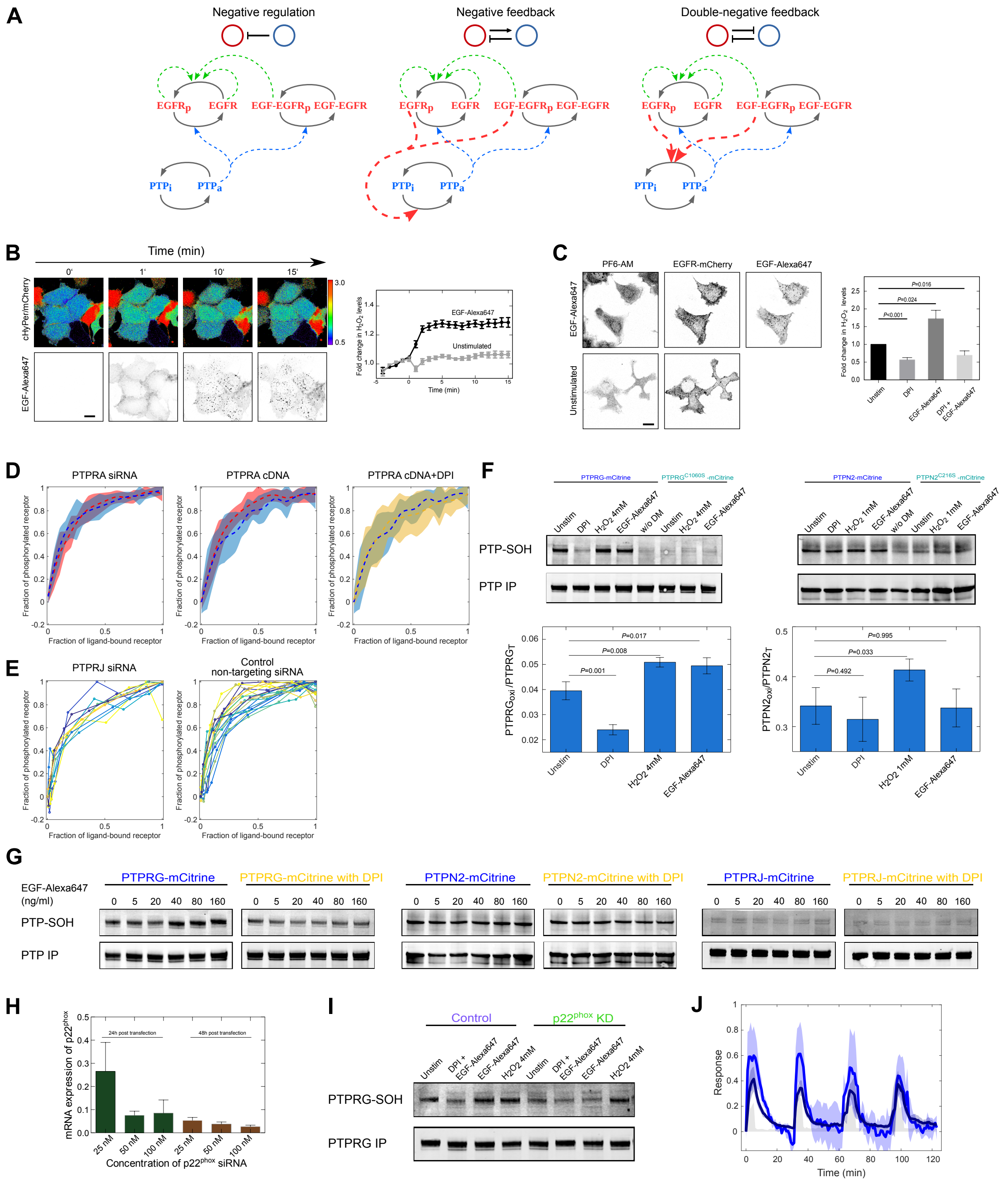

Figure S4. Regulation of EGFR responsiveness by PTPs, Related to Figure 4 and 5. (A) Possible EGFR-PTP network motifs. Solid arrows: molecular state transitions (p: phosphorylation, i: inactive, a: active), dashed arrows: causal links. Left: negative regulation, middle: negative feedback, right: double negative feedback. Top row: corresponding network motifs. **(B)** Left: Representative pseudo-coloured image series of cHyPer3/mCherry fluorescence ratio (upper row), normalized to that at 0min in each MCF7 cell. Images were acquired every minute for 15min after 320ng/ml EGF-Alexa647 stimulation (lower row). Scale bar: 20µm. Right: Corresponding quantification of relative H_2O_2 levels

(cHyPer3/mCherry fluorescence ratio ± SEM) upon EGF-Alexa647 (black line, n=11 cells) or vehicle (unstimulated) administration (grey line, n=9 cells). **(C)** Left: fluorescence images of the H_2O_2-sensitive probe PF6-AM (left), EGFR-mCherry (middle) and EGF-Alexa647 (right) in MCF7 cells with (upper row) and without (lower row) 320ng/ml EGF-Alexa647 stimulation. Scale bar: 20µm. Right: corresponding quantification of H_2O_2 fold-change (mean PF6-AM fluorescence ± SEM from 10 fields of view) upon administration of DPI (10 min, 10µM), EGF-Alexa647 (10 min, 320 ng/ml) or a combination of both. **(D)** Dose-response of EGFR-mTFP phosphorylation (control, red) is not affected upon siRNA-mediated PTPRA knock-down (blue, p=0.823, n=14, N=7, left) or PTPRA-mCitrine co-expression (means±SD, blue, p=0.225, n=34, N=16, middle). NOX-inhibition by DPI (10µM, 30min pre-incubation) has no effect (yellow, p=0.937, n=38, N=16, right) on the dose-response of EGFR phosphorylation upon ectopic PTPRA-mCitrine expression (blue, same as in middle plot). **(E)** Representative dose response curves of EGFR-mTFP phosphorylation in single cells upon siRNA-mediated PTPRJ knock-down (left) and corresponding control with non-targeting siRNA (right). The corresponding means±SD are shown in Figure 4A, bottom. **(F)** Quantification of cysteine oxidation (PTP-SOH) in PTPRG-mCitrine (left) or PTPN2-mCitrine (right) to cysteine sulfenic acid by dimedone conjugation, detected by anti-sulfenic acid modified cysteine antibody. Left: detection of oxidized cysteines in immunoprecipitated PTPRG-mCitrine from MCF7 cells treated for 10 min with DPI (10µM), EGF-Alexa647 (80ng/ml) or 4mM H_2O_2 by western blotting. The weak PTP-SOH bands in the corresponding lanes of the $PTPRG^{C1060S}$-mCitrine mutant show that it is the catalytic cysteine that is oxidized. Right: Oxidation of cysteines in PTPN2-mCitrine and $PTPN2^{C216S}$-mCitrine detected using same conditions as above (means±SEM**,** with the exception of the H_2O_2 dose). **(G)** Cysteine oxidation of PTPRG-mCitrine (left), PTPN2-mCitrine (middle) and PTPRJ-mCitrine (right) upon 10min stimulation with increasing EGF-Alexa647 doses (0-160ng/ml) with and without pretreatment with DPI (20 min, 10µM) (representative WBs). **(H)** mRNA expression of $p22^{phox}$ determined by RT-PCR after 24h or 48h of siRNA transfection with different concentrations (means±SEM**,** N=2). **(I)** Representative WB of cysteine oxidation in PTPRG-mCitrine upon non-targeting (control) or $p22^{phox}$ siRNA transfection. Quantification given in Figure 4E. **(J)** Temporal traces of the fraction of ligand bound (EGF-Alexa647/EGFR-mCitrine, dark) and phosphorylated EGFR estimated by PTB-mCherry translocation to the PM (PTB-mCherry/EGFR-mCitrine, light) in live MCF7 cell following siRNA mediated knock-down of PTPRG (means±SD**,** n=4, N=2). Data was acquired at 1min-intervals following 20ng/ml 5P-EGF every 30min.
