# Supplementary material for: Interdependence between EGFR and Phosphatases Spatially Established by Vesicular Dynamics Generates a Growth Factor Sensing and Responding Network
Source: Cell Syst. 2018 Sep 26;7(3):295–309.e11. doi: 10.1016/j.cels.2018.06.006 (PMC6167251; doi:10.1016/j.cels.2018.06.006)
Supplement: Document S2. Article plus Supplemental Information [file mmc7.pdf]

Cell Systems

Article

Interdependence between EGFR and Phosphatases Spatially Established by Vesicular Dynamics Generates a Growth Factor Sensing and Responding Network

Graphical Abstract

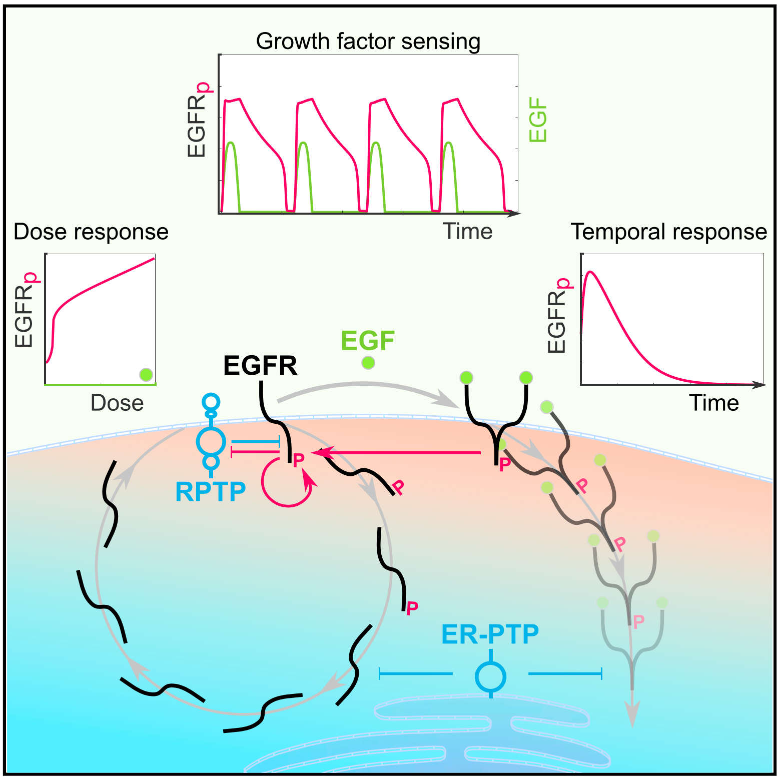

Highlights

- Genetic perturbations reveal three membrane PTPs having highest reactivity for EGFR
- Vesicular dynamics establishes a spatially distributed EGFR-PTP network
- Autocatalytic activation and recursive interactions with PTPs dictate EGFR response
- Dynamical operation mode of this network enables time-varying growth factor sensing

Authors

Angel Stanoev, Amit Mhamane, Klaus C. Schuermann, ..., Jutta Luig, Aneta Koseska, Philippe I.H. Bastiaens

Correspondence

aneta.koseska@mpi-dortmund.mpg.de (A.K.), philippe.bastiaens@mpi-dortmund.mpg.de (P.I.H.B.)

In Brief

Cells continuously respond to temporal changes of growth factors, but it is unclear how the surface receptor tyrosine kinase EGFR senses and translates these changes. We identify three protein tyrosine phosphatases localized on the plasma membrane and the ER that together do more than erase the phosphorylation signal written by this receptor. We show that vesicular recycling unifies the recursive interactions of these phosphatases with autocatalytically activated EGFR, thereby enabling responsiveness to time-varying EGF stimuli.

Stanoev et al., 2018, Cell Systems 7, 295–309
September 26, 2018 © 2018 The Author(s). Published by Elsevier Inc.
https://doi.org/10.1016/j.cels.2018.06.006

OPEN ACCESS
CellPress

Interdependence between EGFR and Phosphatases Spatially Established by Vesicular Dynamics Generates a Growth Factor Sensing and Responding Network

Angel Stanoev,[1,6] Amit Mhamane,[1,6] Klaus C. Schuermann,[1] Hernán E. Grecco,[1,3] Wayne Stallaert,[1] Martin Baumdick,[1] Yannick Brüggemann,[1,2] Maitreyi S. Joshi,[1] Pedro Roda-Navarro,[1,5] Sven Fengler,[1,4] Rabea Stockert,[1] Lisaweta Roßmannek,[1] Jutta Luig,[1] Aneta Koseska,[1,2,*] and Philippe I.H. Bastiaens[1,2,7,*]
[1]Department of Systemic Cell Biology, Max Planck Institute for Molecular Physiology, 44227 Dortmund, Germany
[2]Faculty of Chemistry and Chemical Biology, TU Dortmund, 44227 Dortmund, Germany
[3]Present address: Department of Physics, University of Buenos Aires, 1428 Buenos Aires, Argentina
[4]Present address: Laboratory Automation Technologies, German Center for Neurodegenerative Diseases e.V. of the Helmholtz Society, 53127 Bonn, Germany
[5]Present address: Department of Immunology, Ophthalmology and ORL, Complutense University and "12 de Octubre" Health Research Institute, 28040 Madrid, Spain
[6]These authors contributed equally
[7]Lead Contact
*Correspondence: aneta.koseska@mpi-dortmund.mpg.de (A.K.), philippe.bastiaens@mpi-dortmund.mpg.de (P.I.H.B.)
https://doi.org/10.1016/j.cels.2018.06.006

SUMMARY

The proto-oncogenic epidermal growth factor receptor (EGFR) is a tyrosine kinase whose sensitivity to growth factors and signal duration determines cellular behavior. We resolve how EGFR's response to epidermal growth factor (EGF) originates from dynamically established recursive interactions with spatially organized protein tyrosine phosphatases (PTPs). Reciprocal genetic PTP perturbations enabled identification of receptor-like PTPRG/J at the plasma membrane and ER-associated PTPN2 as the major EGFR dephosphorylating activities. Imaging spatial-temporal PTP reactivity revealed that vesicular trafficking establishes a spatially distributed negative feedback with PTPN2 that determines signal duration. On the other hand, single-cell dose-response analysis uncovered a reactive oxygen species-mediated toggle switch between autocatalytically activated monomeric EGFR and the tumor suppressor PTPRG that governs EGFR's sensitivity to EGF. Vesicular recycling of monomeric EGFR unifies the interactions with these PTPs on distinct membrane systems, dynamically generating a network architecture that can sense and respond to time-varying growth factor signals.

INTRODUCTION

Cells use cell surface receptors such as epidermal growth factor receptor (EGFR) not only to sense the presence of extracellular growth factors but also to interpret the complex dynamic growth factor patterns that can lead to diverse, functionally opposed cellular responses including proliferation, survival, apoptosis, differentiation, and migration (Yarden and Sliwkowski, 2001). Collective EGFR phosphorylation dynamics is thereby the first layer that translates the information encoded in time-varying extracellular growth factor patterns into a cellular outcome. Such a system must have two essential characteristics: sensitivity to non-stationary growth factor inputs and capability to transform these inputs into an intracellular activity pattern that varies in both space and time. However, how this is accomplished on the molecular level remains unclear. Canonically, EGFR activation by growth factors relies on dimerization and allosteric activation of its intrinsic kinase activity, which results in the phosphorylation of tyrosine residues on the C-terminal receptor tail (Arkhipov et al., 2013; Kovacs et al., 2015; Schlessinger, 2002) that serve as docking sites for SH2- or PTB-containing signal transducing proteins (Wagner et al., 2013). A variety of protein tyrosine phosphatases (PTPs) that are expressed at distinct localizations in the cell (Tonks, 2006; Andersen et al., 2001) dephosphorylate EGFR and thereby "erase" the information about the presence of extracellular growth factors that was written in the phosphorylation of the receptor (Lim and Pawson, 2010). However, complex EGFR response dynamics such as those that give rise to robust receptor phosphorylation at a threshold growth factor concentration emerge from recursive interactions with PTPs in combination with autocatalytic receptor activation (Baumdick et al., 2015; Grecco et al., 2011; Koseska and Bastiaens, 2017; Reynolds et al., 2003; Schmick and Bastiaens, 2014; Tischer and Bastiaens, 2003). Even though large-scale studies based on enzymatic assays of purified PTPs (Barr et al., 2009), membrane two-hybrid assays (Yao et al., 2017), and biochemical assays on cell extracts after small interfering RNA (siRNA) knockdown (Tarcic et al., 2009) have identified a number of PTPs that dephosphorylate EGFR (Liu and Chernoff, 1997; Tiganis et al., 1998; Yuan et al., 2010), the dominant PTPs that act in concert with EGFR to determine its collective phosphorylation dynamics remain unknown.

We therefore set out to not only identify these PTPs but also investigate how recursive interactions between these PTPs

This is an open access article under the CC BY license (http://creativecommons.org/licenses/by/4.0/).

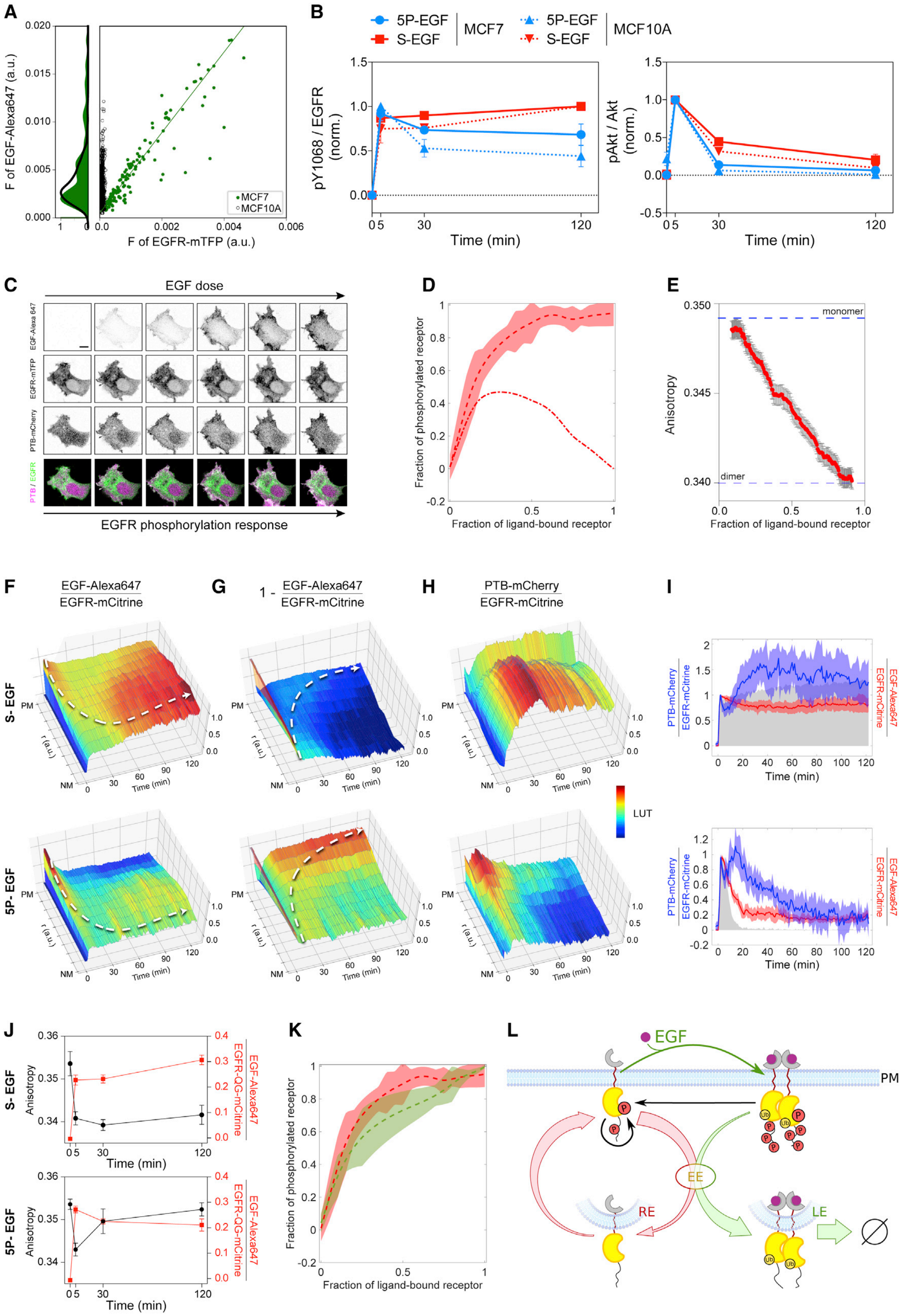

(legend on next page)

and EGFR are established. We specifically asked whether there is a core EGFR-PTP network that determines the receptor's phosphorylation dynamics in response to non-stationary growth factor patterns. To first understand how the interaction of EGFR with PTPs is spatially regulated, we assessed how the phosphorylation of EGFR relates to its vesicular trafficking. We then combined reciprocal and quantifiable genetic PTP perturbations with single-cell quantitative imaging of EGFR to find the strongest EGFR dephosphorylating activities. Spatial-temporal analysis of EGFR phosphorylation upon reciprocal genetic PTP perturbations revealed how EGFR signal duration is regulated, whereas single-cell dose-response experiments demonstrated how EGFR responsiveness to EGF arises. Experimentally supported dynamical systems analysis showed that vesicular dynamics unifies the recursive interactions between EGFR and PTP receptor types (PTPRs) at the plasma membrane with PTPN2 on the ER to enable sensing of, as well as robust activation upon time-varying EGF stimuli.

RESULTS

Ligandless and Liganded EGFR Exhibit Distinct Vesicular and Phosphorylation Dynamics

To investigate how PTPs determine EGFR's response to growth factors, we first assessed how the phosphorylation of EGFR relates to EGF dose and its vesicular trafficking. Fluorescently tagged EGFR-mTFP was ectopically expressed in breast cancer-derived MCF7 cells with low endogenous EGFR expression ($\sim 10^3$/cell [Charafe-Jauffret et al., 2005], Figure S1A), to a level that fell within the endogenous EGFR expression range of the related MCF10A cells (determined by EGF-Alexa647 binding, Figure 1A). EGFR-mTFP expressing MCF7 cells exhibited equivalent EGFR phosphorylation- (Y_{1068}-Grb2 binding site [Okutani et al., 1994]) and Akt activation dynamics to MCF10A cells in response to 200 ng/mL sustained (S-EGF) as well as 5-min pulsed (5P-EGF) EGF-Alexa647 stimulus (Figure 1B). This suggests that EGFR-mTFP-expressing MCF7 cells exhibit physiological EGFR response properties.

To first assess the sensitivity of EGFR phosphorylation response to EGF binding, we performed single-cell dose-response experiments with fluorescent EGF-Alexa647 (Figure 1C). To deconvolute EGF binding kinetics from EGFR's response, we directly related the fraction of liganded receptors to EGFR phosphorylation, which is not possible by analytical biochemical approaches on cell extracts. The fraction of liganded EGFR-mTFP at the plasma membrane was determined by EGF-Alexa647/EGFR-mTFP, and EGFR-mTFP phosphorylation was measured by the rapid translocation of mCherry-tagged phosphotyrosine-binding domain (PTB-mCherry, Figure S1E) to the phosphorylated tyrosines 1086/1148 of EGFR at the plasma membrane (Offterdinger et al., 2004) (Figures S1B–S1E and STAR Methods).

The observed steep EGFR phosphorylation response (Figures 1D and S1D) showed that the largest fraction of phosphorylated receptors at low EGF doses are ligandless (dash-dotted line in Figure 1D; STAR Methods), pointing to an amplification of ligandless EGFR phosphorylation that contributes to this steepness. The high fraction of phosphorylated EGFR at low fraction of liganded receptors additionally indicates that liganded EGFR triggers the phosphorylation amplification on ligandless EGFR. Measuring the dimerization state of EGFR as function of EGF dose by homo-FRET (Förster resonance energy transfer) detection with fluorescence anisotropy microscopy on a fully active EGFR-QG-mCitrine construct (Baumdick et al., 2015), showed that the fraction of ligand-bound receptors corresponds to the fraction of dimerized EGFR (Figures 1E, S1F, and S1G). From this, it can be deduced that phosphorylated ligandless EGFR is monomeric.

Figure 1. EGFR Phosphorylation and Vesicular Dynamics

(A) Quantifying ectopic EGFR-mTFP expression in MCF7 cells. Average EGF-Alexa647 versus EGFR-mTFP fluorescence in single MCF7 (green) or MCF10A cells without EGFR-mTFP (black). Histograms (left) reflect that levels of EGF-Alexa647 binding to MCF7 with ectopic EGFR-mTFP expression (green) and MCF10A with endogenous EGFR (black) are similar.

(B) EGFR Y_{1068} phosphorylation (left) and Akt phosphorylation (right) in MCF7 cells ectopically expressing EGFR-mTFP (solid lines) and for endogenous EGFR in MCF10A cells (dashed lines), following 5-min pulsed (5P-EGF, 200 ng/mL, blue) or sustained EGF stimulation (S-EGF, 200 ng/mL, red), determined by in-cell western assay. Data are normalized to the maximum response in each respective condition (means ± SEM, N = 3).

(C) Representative fluorescence image series of EGF-Alexa647, EGFR-mTFP, PTB-mCherry, and PTB-mCherry (magenta)/EGFR-mTFP (green) overlay from single-cell dose-response experiment. Cells were stimulated every ~1.5 min with increasing EGF-Alexa647 doses (2.5–600 ng/mL). Scale bar, 20 μm.

(D) Fraction of phosphorylated versus ligand-bound EGFR-mTFP (n = 21, N = 10; Figures S1B–S1D). Dashed lines: moving averages from single cells; shaded bounds: SDs; dash-dotted lines: estimated contribution of ligandless to the fraction of phosphorylated EGFR.

(E) Live cell fluorescence anisotropy microscopy measurements of EGFR-QG-mCitrine dimerization state as a function of the fraction of ligand-bound receptor (mean ± SEM, n = 30, N = 3, Figures S1F and S1G).

(F–H) Average spatial-temporal maps of the estimated fraction of ligand-bound EGFR (F, EGF-Alexa647/EGFR-mCitrine), ligandless EGFR (G, 1 − [EGF-Alexa647/EGFR-mCitrine]), and the fraction of phosphorylated EGFR-mCitrine estimated by PTB-mCherry translocation (H, PTB-mCherry/EGFR-mCitrine). Data were acquired at 1-min intervals in live MCF7 cells following 200 ng/mL S-EGF (top, n = 16, N = 3; Figures S1I and S1J) or 5P-EGF (n = 14, N = 2; Figures S1I and S1J) stimulation. White dotted lines: trajectories representing the change in distribution of ligand-bound (F) and ligandless (G) EGFR. PM, plasma membrane; NM, nuclear membrane.

(I) The respective plasma membrane fractions of ligand-bound (EGF-Alexa647/EGFR-mCitrine, red) and phosphorylated EGFR (PTB-mCherry/EGFR-mCitrine, blue) derived from (F) and (H) (median ± AMD). Extracellular EGF-Alexa647 fluorescence is shown in gray.

(J) Dimerization state measured by anisotropy (black) and the fraction of ligand-bound EGFR-QG-mCitrine (red) at the plasma membrane for live cells following 200 ng/mL S-EGF (top, n = 5, N = 3) or 5P-EGF (bottom, n = 5, N = 3) stimulation (means ± SEM).

(K) The dose response of EGFR-mTFP phosphorylation (red, control) is significantly altered upon ectopic $Rab11^{S25N}$ expression (green; p = 0.02; n = 12, N = 4). Lines are the same as in (D).

(L) EGFR trafficking dynamics: ligandless EGFR recycles via early (EE) and recycling endosomes (RE) to the plasma membrane (red arrows) whereas upon EGF binding (thin green arrow), ubiquitinated $EGF\text{-}EGFR_{Ub}$ unidirectionally traffics via the early to the late endosomes (LE, green arrow) to be degraded in lysosomes (∅). Causal links are denoted by solid black lines.

Given the contribution of ligandless monomers to the sensitivity of EGFR activation, we investigated how vesicular dynamics relates to EGFR phosphorylation by exposing cells to both sustained (S-EGF) and pulsed (5P-EGF) stimulation. For this, EGFR-mCitrine, EGF-Alexa647, and PTB-mCherry fluorescence distributions were monitored by live cell confocal microscopic imaging, and receptor self-association was monitored in a separate experiment by fluorescence anisotropy microscopy on EGFR-QG-mCitrine. The molecular quantities of ligandless EGFR fraction at each pixel was calculated from 1 − [EGF-Alexa647/EGFR-mCitrine], and EGFR phosphorylation from PTB-mCherry/EGFR-mCitrine. The radial symmetry in receptor trafficking from the plasma membrane to the nuclear membrane enabled dimensionality reduction of the Cartesian variables (x, y) to normalized radial variable (r, Figure S1H), which allowed us to reconstruct average 3D spatial-temporal maps from multiple cells (Figures 1F–1H, S1I, and S1J; STAR Methods).

Upon sustained EGF stimulation, liganded dimers (Figure 1F [top], EGF-Alexa647/EGFR-mCitrine) at the plasma membrane were activated (Figures 1H and 1I [top], PTB-mCherry/EGFR-mCitrine), endocytosed, and unidirectionally trafficked toward the perinuclear area in the course of 2 hr, where they were inactivated by dephosphorylation (Figures 1H and S1I; Video S1). Retrograde trafficking of ligandless receptors from the perinuclear recycling endosome to the plasma membrane (Baumdick et al., 2015) was also observed following S-EGF stimulation (Figure 1G, top), where they immediately bound EGF. This was reflected in the continuous high fraction of dimers at the plasma membrane, as measured by the anisotropy of EGFR-QG-mCitrine (Figure 1J, top).

To next investigate if receptors can autophosphorylate after a stimulus is removed, we exposed cells to a 5-min pulse of EGF (5P-EGF) and spatially resolved EGFR's phosphorylation dynamics over 2 hr. During the pulse, receptors bound EGF and were depleted from the plasma membrane to accumulate in the perinuclear area, where they were dephosphorylated (Figures 1F and 1H, bottom). However, in the time after the pulse, ligandless receptors rapidly recycled to the plasma membrane ($t_{1/2}$ ~4 min, STAR Methods; Figures 1G [bottom] and S1K; Video S2) where they were rephosphorylated in the absence of ligand, exhibiting their maximal phosphorylation at ~15 min after 5P-EGF to then slowly decay to a dephosphorylated state (Figure 1I, bottom). Fluorescence anisotropy measurements of EGFR-QG-mCitrine showed that the recycled EGFR was monomeric (Figure 1J, bottom). In accordance with this, the blocking of vesicular recycling by ectopic expression of dominant negative Rab11^{S25N} mutant (Konitsiotis et al., 2017) led to a significant decrease in the steepness of the EGFR phosphorylation response (Figure 1K).

These experiments thus show that ligandless and liganded EGFR exhibit distinct vesicular and phosphorylation dynamics that can be distinguished by 5P-EGF stimulus. Upon ligand binding, ligandless EGFR is transformed to dimeric EGFR (green arrow, Figure 1L). The dimers can in turn activate autophosphorylation on remaining or recycling monomeric EGFR (black arrow, Figure 1L), thereby amplifying the response. In contrast to the recycling ligandless monomeric EGFR, which can additionally be reactivated by autocatalysis at the plasma membrane (Baumdick et al., 2015), liganded dimeric EGFR unidirectionally traffics to late endosomes. This indicates that a continuously maintained fraction of EGFR monomers at the plasma membrane allows for sensing of upcoming growth factor stimuli.

The Major PTPs that Dephosphorylate EGFR Are on the ER and the Plasma Membrane

To investigate how PTPs regulate EGFR phosphorylation in this vesicular dynamic system, we identified which PTPs have the strongest non-redundant dephosphorylating activity on EGFR. For this, it was necessary to apply reciprocal genetic perturbations of siRNA-mediated knockdown of a given PTP (PTP_X) as well as ectopic expression of fluorescently tagged PTP_X-mCitrine. siRNA-mediated PTP_X knockdown reveals non-redundant PTPs that regulate EGFR-EGFP phosphorylation, but neither the strength of enzymatic activity toward phosphorylated EGFR nor direct or indirect regulation can be derived. On the other hand, ectopic co-expression of PTP_X-mCitrine isolates its negative regulatory effect on the EGFR-mTFP phosphorylation cycle from endogenous PTPs. With this perturbation approach, EGFR phosphorylation can be related to the magnitude of PTP_X-mCitrine expression in each cell to derive a measure of specific phosphatase activity.

The change in EGFR phosphorylation in response to these reciprocal genetic perturbations was measured by determining the change in FRET that occurs upon binding of an anti-phosphotyrosine antibody tagged with Cy3.5 (PY72-Cy3.5) to phosphorylated EGFR fused to a fluorescent protein (EGFR-FP [Wouters and Bastiaens, 1999]). FRET was measured via the decrease in fluorescence lifetime of EGFR-FP in single cells using cell-array fluorescence lifetime imaging microscopy (CA-FLIM), and the fraction of phosphorylated EGFR-FP (α) was quantified using global analysis (Grecco et al., 2010) (Figures S2A and S2B). The effect of the genetic PTP perturbations on EGFR phosphorylation was then determined by the phosphorylation fold change (PFC): $PFC_\alpha = \alpha_{PTP}/\alpha_{ctr}$.

CA-FLIM screening of 55 PTPs that are expressed in MCF7 cells (Figure S1A; Tables S2 and S3) and well represent the four PTP families (Alonso et al., 2004), showed that 39 significantly affected EGFR phosphorylation (PFC_α) after 5 min of EGF stimulation. However, only 5 PTPs increased EGFR phosphorylation upon knockdown (PFC_α − siRNA) and decreased it upon ectopic PTP_X-mCitrine expression (PFC_α − cDNA), identifying them as non-redundant negative regulators of EGFR phosphorylation (Figure 2A, red dots in quadrant 1, diameter proportional to mRNA expression in MCF7 cells). These were the ER-bound PTPN2 (Lorenzen et al., 1995) and the receptor-like PTPR-G/J/A (Andersen et al., 2001; Barr et al., 2009) belonging to the family of classical PTPs, as well as the dual-specificity phosphatase DUSP3. Additionally, the lowly expressed DUSP7 and DUSP10 were identified as positive regulators with both genetic perturbations (Figure 2A, red dots in quadrant 3). These are necessarily indirect effectors, implicating that the expression level of auxiliary proteins does not limit their positive regulation of EGFR phosphorylation.

Most of the remaining 32 PTPs affected EGFR phosphorylation only upon knockdown (PFC_α − siRNA, blue lines, Figure 2A), whereas 6 had an effect only upon ectopic expression (PFC_α − cDNA, green lines, Figure 2A). The majority of these PTPs fell on the right of the cDNA axis and below the siRNA axis, and

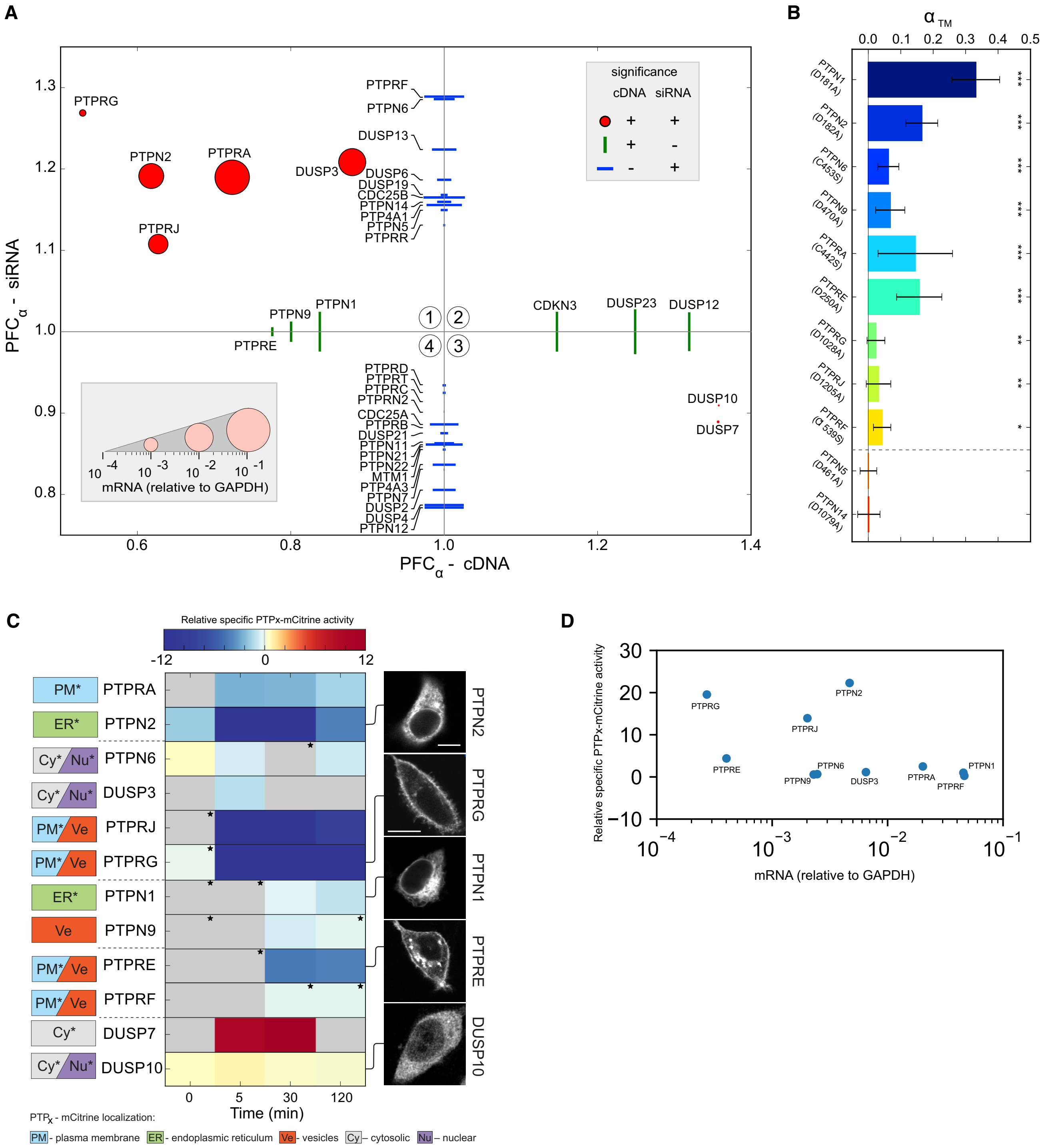

Figure 2. *In Situ* EGFR Phosphatome Identification

(A) Scatterplot of median EGFR phosphorylation fold changes ($PFC_\alpha = \alpha_{PTP}/\alpha_{ctr}$, n ~ 150 cells per condition) upon siRNA knockdown (PFC_α – siRNA) and ectopic PTP$_X$-mCitrine expression (PFC_α – cDNA), 5 min after 200 ng/mL EGF stimulation. Significant PFC_α upon both (red dots) or only one perturbation (green/blue lines, $p < 0.05$) are shown. Marker length is scaled to relative PTP$_X$-mRNA expression in MCF7 cells (legend: bottom inset and Figure S1A).

(B) Average fraction of EGFR-mTFP interacting with catalytically impaired PTP$_X$-mCitrine trapping mutants (α_{TM} ± SD, n = 15–20 cells, N = 2; *$p < 0.05$, **$p < 0.01$, ***$p < 0.001$) following 200 ng/mL 5P-EGF.

(C) Relative specific PTP$_X$-mCitrine activities prior to and 5, 30, and 120 min after 200 ng/mL 5P-EGF stimulation (middle). Asterisks denote weak linear dependencies (Figure S2D). Subcellular localization of PTP$_X$-mCitrine (asterisks in left-hand boxes: additionally curated localization from LOCATE/UniProt database) and exemplary fluorescence images (right; scale bars, 10 μm).

(D) Relative specific PTP$_X$-mCitrine activities versus the corresponding mRNA expression in MCF7 cells.

OPEN ACCESS CellPress

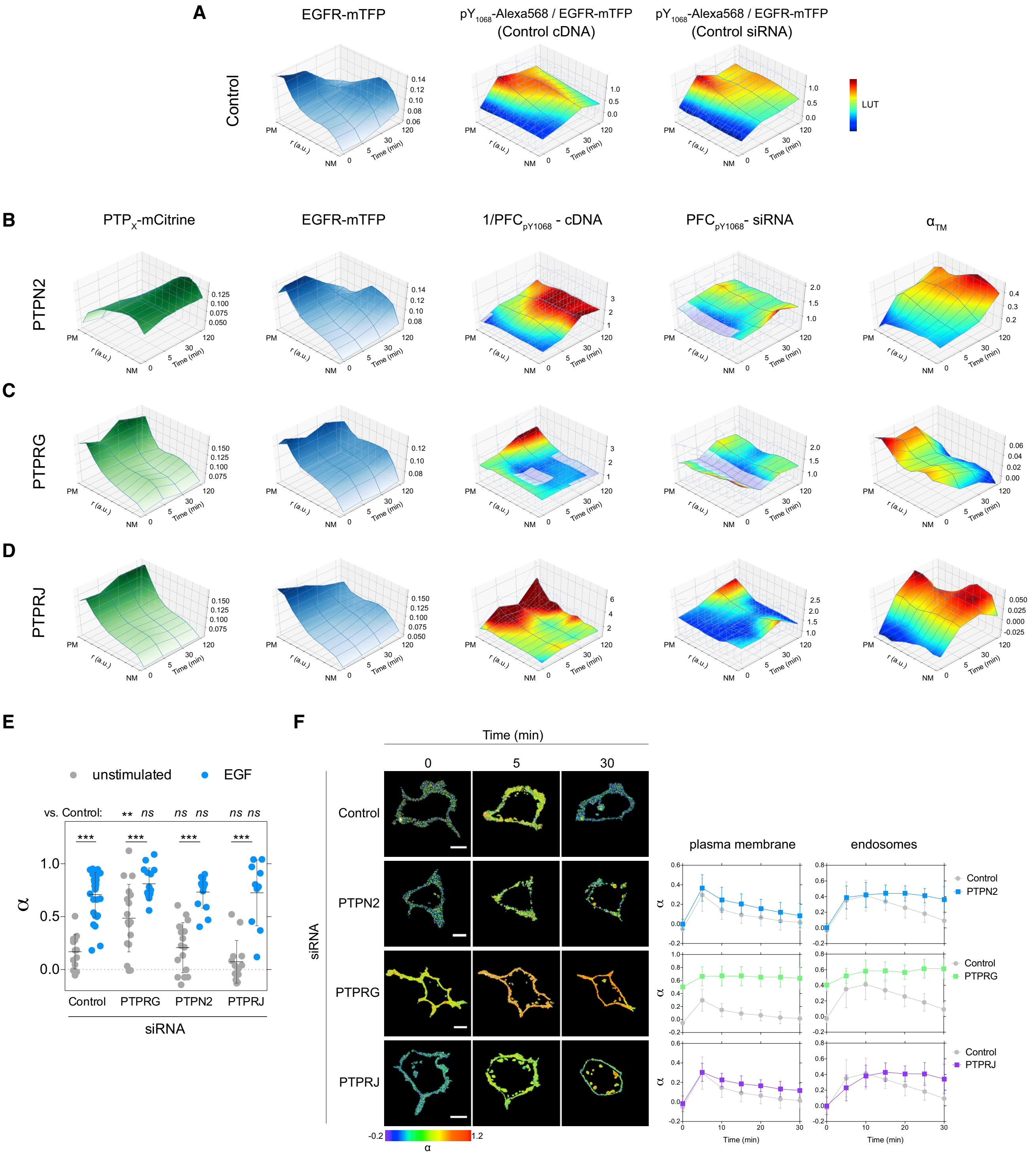

Figure 3. Spatial-Temporal Regulation of EGFR Phosphorylation by PTPN2 and PTPRG/J

(A) Spatial-temporal maps (STMs) depicting EGFR-mTFP fluorescence (left) and pY_{1068} phosphorylation (middle) in control cells (n ~ 90 cells per time point for a total of ~360 cells, N = 6 experiments) and following transfection with non-targeting siRNA pool (right, n ~ 60, N = 4).

(B) Columns 1–3: effect of PTPN2-mCitrine expression (column 1) on STMs of EGFR-mTFP localization (column 2) and phosphorylation fold change ($1/PFC_{pY1068}$-cDNA, column 3) (n ~ 60, N = 3). Column 4: effect of siRNA-mediated PTPN2 knockdown on EGFR-mTFP phosphorylation fold change (PFC_{pY1068}-siRNA, n ~ 45, N = 3). Column 5: STM of fraction of EGFR-mTFP interacting with $PTPN2^{C216S}$-mCitrine trapping mutant as determined by FLIM (α_{TM}, n = 15–30, N = 2).

(C) STMs of the same quantities as in (B) upon PTPRG-mCitrine expression/siRNA-mediated knockdown (n ~ 60, N = 3; α_{TM} $PTPRG^{C1060S}$-mCitrine n = 15–30, N = 2).

(legend continued on next page)

are therefore indirect positive regulators of EGFR phosphorylation. On the other hand, the effect of the negative regulators that manifests only upon a single genetic perturbation reflects either redundancy in the case of ectopic expression, or PTPs whose activity depends on and is limited by the amount of phosphorylated EGFR in the case of knockdown.

FLIM-FRET measurements of the interaction of EGFR-mTFP with fluorescent, catalytically impaired PTP trapping variants (Flint et al., 1997) showed that the four classical non-redundant (PTPN2, PTPRG/J/A) (Figure 2B and Table S3) and the redundant negative regulators (PTPN1/9, PTPRE; identified upon ectopic expression), as well as the strongest negative regulators identified upon knockdown (PTPN6, PTPRF) directly dephosphorylate EGFR. On the other hand, interaction with EGFR-mTFP was not observed with the trapping variants of indirect negative regulators (PTPN5, PTPN14) (Belle et al., 2015; José et al., 2003).

To determine which of the identified PTPs exert the strongest dephosphorylating activity on EGFR, we used cell-to-cell variance in PTP_X-mCitrine expression to estimate the specific activity of each of these PTPs toward EGFR-mTFP. For this, we measured EGFR-mTFP phosphorylation (α_t, Figure S2C) and ectopic PTP_X-mCitrine expression in individual cells upon 5P-EGF to generate scatterplots of the fraction of phosphorylated EGFR (α) versus PTP_X-mCitrine fluorescence for a given time point (Figure S2D). The scatterplots were parameterized by an exponential fit to obtain the specific activities. This showed that three of the non-redundant negative regulators identified from the reciprocal perturbations (PTPN2 and PTPRG/J) exhibited the strongest dephosphorylating activity toward EGFR-mTFP that extended over the full time range after EGF stimulation (Figures 2C and 2D). These three strongest regulators are associated with distinct membrane systems whereby PTPN2 is bound to the cytoplasmic face of the ER and PTPRG/J are on the plasma membrane (Figure 2C). PTPRJ/G exhibited one to two orders of magnitude lower mRNA expression than PTPN2 (Figure 2D), which points at an overall lower PTP activity at the plasma membrane as compared with the cytoplasm. In contrast, the highly expressed soluble DUSP3 and plasma membrane localized PTPRA were profiled as only weak or modest regulators of EGFR phosphorylation, respectively.

PTPN2 and PTPRs Dynamically Shape EGFR's Phosphorylation Response in Space

To determine how the juxtaposed PTPs shape EGFR phosphorylation in space, we imaged the effect of reciprocal genetic PTP_X perturbations on Y_{1068} EGFR-mTFP phosphorylation after 5P-EGF in many individual cells by immunofluorescence using a specific pY_{1068} antibody (Figures S3A and S3B). From these images, we reconstructed 3D spatial-temporal maps of the average fraction of phosphorylated EGFR-mTFP (pY_{1068}-Alexa568/EGFR-mTFP; Figure 3A and STAR Methods) at 0, 5, 30, and 120 min following 5P-EGF stimulation. To map where the PTP_X dephosphorylates EGFR-mTFP, we quantified the genetic perturbation effects by the PFC relative to control (ctr) defined by

$$PFC_{pY1068} - siRNA_X = \frac{\left(\frac{pY_{1068} - Alexa568}{EGFR - mTFP}\right)_{PTP_X}}{\left(\frac{pY_{1068} - Alexa568}{EGFR - mTFP}\right)_{ctr}}$$

for knockdown of a PTP_X (Figure S3C), and

$$1/PFC_{pY1068} - cDNA_X = \frac{\left(\frac{pY_{1068} - Alexa568}{EGFR - mTFP}\right)_{ctr}}{\left(\frac{pY_{1068} - Alexa568}{EGFR - mTFP}\right)_{PTP_X}}$$

for ectopic PTP_X-mCitrine expression. Average PFCs of many cells were accumulated using the same dimensionality reduction and distance normalization as in Figures 1F–1H.

Mathematical modeling of the phosphorylation/dephosphorylation cycle showed that the experimentally derived $1/PFC_{pY1068} - cDNA_X$ approximates the local specific dephosphorylating activity of an ectopically expressed PTP_X-mCitrine relative to the local kinase activity of EGFR ($1/PFC_{pY1068} - cDNA_X \approx k_{ptpx}\,[PTP_X]/k_{EGFR}$; STAR Methods). To avoid loss of spatial information on these activities due to PTP_X-mCitrine overexpression-induced saturation, we only analyzed cells where pY_{1068}/EGFR depended linearly on PTP_X-mCitrine (Figure S3D).

The spatial-temporal map of PTPN2-mCitrine fluorescence shows that PTPN2 concentration steadily declines from the perinuclear area toward the cell periphery, invariant over time (Figure 3B, PTP_X-mCitrine), whereas the profile of fluorescent EGFR-mTFP reflected the typical vesicular dynamic behavior of internalization and recycling after 5P-EGF (Figure 3B, EGFR-mTFP). Thus, as phosphorylated EGFR traffics from the plasma membrane via early to late or recycling endosome along this increasing PTPN2 concentration, it is progressively dephosphorylated on pY_{1068} (Figure 3B, PFCs). Both $PFC_{pY1068} - siRNA_{PTPN2}$ and $1/PFC_{pY1068} - cDNA_{PTPN2}$ additionally showed an increasing dephosphorylating activity of PTPN2 with time at the cell periphery, revealing that a minor fraction of ER-bound PTPN2 can reach the plasma membrane (Lorenzen et al., 1995) to dephosphorylate EGFR-pY_{1068}. This was corroborated by the interaction profile of EGFR-mTFP with the trapping

(D) STMs of the same quantities as in (B) upon PTPRJ-mCitrine expression/siRNA-mediated knockdown ($n \sim 40$, N = 2; α_{TM} $PTPRJ^{D1205A}$-mCitrine, $n \sim 30$, N = 2). In (A) to (D), cells were stimulated with 200 ng/mL 5P-EGF; transparent areas denote non-significant PFCs, $p > 0.05$.

(E) Effect of siRNA-mediated knockdown of PTPRG, PTPN2, and PTPRJ on the fraction of phosphorylated EGFR (α) in single MCF7 cells expressing EGFR-mCitrine (donor) and PTB-mCherry (acceptor). FLIM measurements were made prior to (gray) and 2 min after saturating 320 ng/mL EGF-Alexa647 stimulation (blue). α_{mean} ± SD for control: n = 14 (gray), n = 17 (blue); PTPRG: n = 15 (gray), n = 11 (blue); PTPN2: n = 9 (gray), n = 8 (blue); PTPRJ: n = 6 (gray), n = 6 (blue). N = 1–2. **p = 0.0018 and ***p < 0.001; ns, not significant.

(F) Time-lapse measurements of the fraction of phosphorylated EGFR (as above) in single MCF7 cells prior to and every 5 min after 200 ng/mL 5P-EGF stimulation for a total of 30 min. Representative α images (left) and corresponding quantifications (right) for control (n = 4), PTPN2 (n = 5), PTPRG (n = 5), and PTPRJ (n = 4) knockdowns (N = 3, means ± SD). Scale bars, 10 μm.

PTPN2^{C216S}-mCitrine variant (Tiganis et al., 1998), which increased both toward the perinuclear and the peripheral cytoplasm over time (Figure 3B, α_{TM}).

PTPRG-mCitrine displayed strong fluorescence at the cell periphery that abruptly declined in the cytoplasm, but in contrast to PTPN2 exhibited dynamic redistribution after stimulation (Figure 3C, PTP$_X$-mCitrine). This redistribution of PTPRG coincided with that of EGFR (Figure 3C, EGFR-mTFP), initially internalizing in endosomes, to then traffic back and gradually increase at the plasma membrane. This points at a direct interaction of PTPRG and EGFR. The $PFC_{pY1068} - siRNA_{PTPRG}$ showed an enhanced phosphorylation of EGFR in the absence of stimulus, indicating that PTPRG maintains EGFR monomers dephosphorylated. After 5P-EFG, both $PFC_{pY1068} - siRNA_{PTPRG}$ and $PFC_{pY1068} - cDNA_{PTPRG}$ revealed a steady increase in PTPRG activity at the plasma membrane over time (Figure 3C).

PTPRJ-mCitrine distribution did not coincide with that of EGFR, translocating from endosomes back to the plasma membrane late after 5P-EGF (Figure 3D, compare PTP$_X$-mCitrine with EGFR-mTFP). In stark contrast to PTPRG, the dephosphorylating activity of PTPRJ was low in the absence of stimulus and increased after 5P-EGF, following its observed redistribution to the plasma membrane. The differences in the interaction of EGFR-mTFP with the trapping variants of the two PTPRs (α_{TM}, Figures 3C and 3D) reflect their differences in regulating EGFR dephosphorylation. Whereas the interaction of the PTPRG^{C1060S}-mCitrine (Table S3) with EGFR-mTFP already occurred in the absence of stimulus (α_{TM} 0 min, Figure 3C), the interaction with PTPRJ^{D1205A}-mCitrine was apparent and increasing only after 5P-EGF (α_{TM}, Figure 3D). This indicates that PTPRG preferentially dephosphorylates ligandless EGFR at the plasma membrane, corroborated by the strongly reduced PTPRG activity upon S-EGF stimulus when the majority of the receptor is liganded (Figure S3E). Immunoprecipitation experiments further confirmed that there is a preferential interaction of PTPRG-mCitrine with ligandless EGFR (activated due to PTP inhibition by H_2O_2 [Meng et al., 2002]) over liganded EGFR activated by EGF, whereas PTPRJ constitutively interacts with both species (Figure S3H). The spatial-temporal map α_{TM} of PTPRJ^{D1205A}-mCitrine also showed an increase of interaction in the perinuclear cytoplasm after 5P-EGF, which is consistent with the $PFC_{pY1068} - siRNA_{PTPRJ}$ and indicates that an intracellular endosomal fraction of PTPRJ dephosphorylates endocytosed EGFR.

The more static spatial-temporal distribution of the other identified non-redundant receptor-like PTPRA-mCitrine did not coincide with that of EGFR (Figure S3F, compare PTP$_X$-mCitrine with EGFR-mTFP). Even more, its specific activity toward EGFR-pY$_{1068}$ increased at intermediate and late times after EGF stimulation (Figure S3F, $PFC_{pY1068} - siRNA_{PTPRA}$, $1/PFC_{pY1068} - cDNA_{PTPRA}$), following the interaction profile of EGFR-mTFP with the trapping PTPRA^{C442S}-mCitrine variant (Figure S3F, α_{TM}). This indicates that PTPRA suppresses autonomous activation of recycling ligandless receptors mostly at the plasma membrane late after stimulus. siRNA-mediated knockdown of DUSP3 confirmed the low specific activity (Figure 2C) of this atypical phosphatase toward EGFR-pY$_{1068}$ (Figure S3G).

To further investigate how the three strongest PTPs affect EGFR phosphorylation dynamics, we measured time-lapse EGFR-mTFP phosphorylation response to 5P-EGF in living cells upon PTP$_X$ knockdown. EGFR phosphorylation was imaged via the interaction of PTB-mCherry with phosphorylated EGFR-mCitrine by FLIM and quantified by global analysis (Grecco et al., 2010) to obtain the average fraction of phosphorylated EGFR (α) at the plasma membrane and on endosomes (Figures 3E and 3F; STAR Methods). PTPRG knockdown resulted in substantially elevated basal EGFR phosphorylation (Figure 3E), in line with its trapping variant already interacting with EGFR in the absence of stimulus (Figure 3C). The wide distribution of EGFR phosphorylation in this case likely reflects the variance in PTPRG knockdown level in each cell. Consistently, time-lapse FLIM of EGFR phosphorylation showed the already high EGFR phosphorylation on the plasma membrane and in endosomes in the absence of stimulus to only slightly increase to a plateau after 5P-EGF (Figure 3F). PTPRJ knockdown resulted in more sustained phosphorylation of EGFR monomers at the plasma membrane after 5P-EGF. We observed a steady increase in the phosphorylation on endosomes that plateaued 15 min after 5P-EGF. This indicates that PTPRJ dephosphorylates recycling EGFR monomers. In contrast, PTPN2 knockdown only changed the amplitude of the response at the plasma membrane without affecting its profile, whereas activation of EGFR signaling from endosomes initially followed that at the plasma membrane but was then clearly sustained at later times.

These results are consistent with the PFCs (Figures 3B–3D) and show that PTPN2 determines signal duration by dephosphorylating liganded EGFR during its vesicular trafficking, whereas PTPRG and PTPRJ dephosphorylate recycling ligandless EGFR. This suggests that PTPRG/J most likely have a functional role in determining the sensitivity of EGFR phosphorylation response to EGF.

PTPRG Is a Central Regulator of EGFR Responsiveness to EGF Dose

To understand how EGFR sensitivity to growth factors is regulated by the distinct activity of PTPRG/J at the plasma membrane and PTPN2 on the ER, we determined EGFR-mTFP phosphorylation response to EGF dose upon PTP$_X$ knockdown. This was performed in single cells analogous to the experiments presented in Figures 1C and 1D. The pre-activation of EGFR phosphorylation upon PTPRG knockdown (Figures 3E and 4A [top]) impedes EGFR responsiveness to EGF, and we therefore did not perform this experiment. PTPRJ knockdown induced a more switch-like EGFR phosphorylation response (Figures 4A [bottom] and S4E), whereas knockdown of PTPN2 significantly steepened the EGF dose response (Figure 4A, middle). Knockdown of PTPRA did not affect the EGF dose-EGFR phosphorylation response (Figure S4D), consistent with its late function in suppressing autonomous activation of recycling receptors at the plasma membrane (Figure S3F).

These PTP$_X$ knockdown experiments do not allow to derive the causality between PTP$_X$ and EGFR that underlie EGFR's response to EGF. For this a positive perturbation is necessary, which was imposed by ectopic expression of PTP$_X$-mCitrine. To infer the causality relation between PTP$_X$ and EGFR, we fitted the models for the three possible modes of interaction—negative regulation, negative feedback, and double-negative feedback (Figure S4A and STAR Methods)—to the dose-response curves.

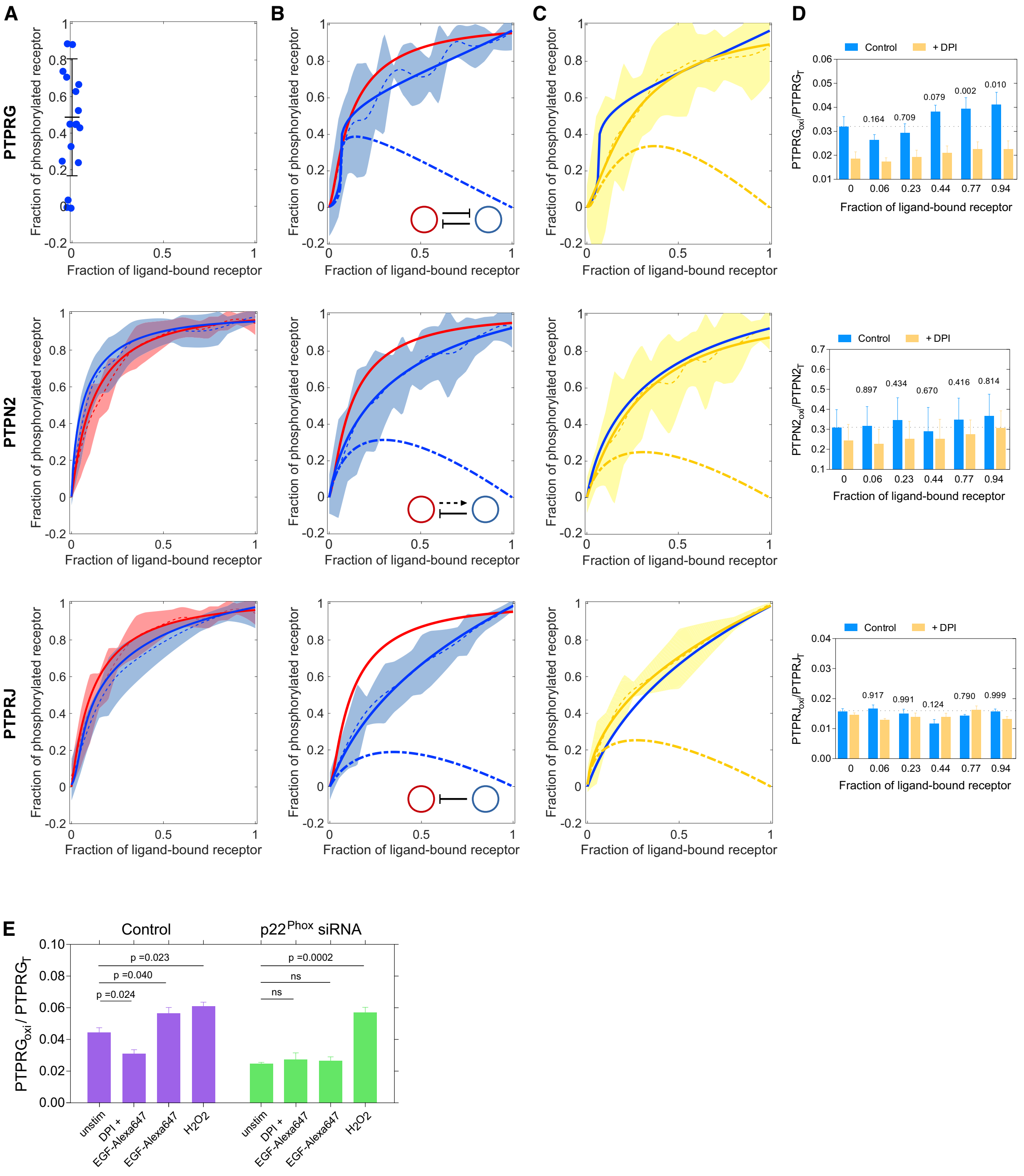

Figure 4. Differential Regulation of EGFR Responsiveness by PTPN2 and PTPRs

(A) Averaged single-cell dose-response measurements following PTP_X knockdown. PTPRG knockdown results in EGFR phosphorylation in absence of stimulus (top, blue dots on the y axis as in Figure 3E). Dose response of EGFR-mCitrine phosphorylation (red, n = 21, N = 6) is significantly altered upon siRNA-mediated PTPRJ knockdown (bottom, blue line, p = 0.004; n = 11, N = 3) and less upon PTPN2 knockdown (middle, blue line, p = 0.17; n = 14, N = 6). Shaded bounds as in Figure 1D. Solid lines: model-based fits to the phosphorylated EGFR fraction (STAR Methods and Figure S4A).

(B) Dose response of EGFR-mTFP phosphorylation (red) is significantly altered upon PTPRG-mCitrine co-expression (blue lines, n = 28, N = 14, p = 0.027; top), PTPN2-mCitrine (blue lines, n = 34, N = 13, p = 0.001; middle), or PTPRJ-mCitrine co-expression (n = 16, N = 7, $p = 4 \times 10^{-4}$; bottom). Solid lines: model-based fits to the phosphorylated EGFR fraction (STAR Methods and Figure S4A). Best fits are with the model shown in the inset.

(C) NOX inhibition by DPI (10 μM, 30 min pre-incubation) significantly flattens dose response of EGFR phosphorylation upon ectopic PTPRG-mCitrine (top, yellow lines, p = 0.06; n = 26, N = 10), but has no effect upon PTPN2-mCitrine (middle, p = 0.19; n = 45, N = 12) or PTPRJ-mCitrine expression (bottom, p = 0.162; n = 10, N = 5).

(legend continued on next page)

The model that best fitted the data was selected using the Akaike information criterion (STAR Methods and Table S1). A switch-like EGFR phosphorylation response was observed upon ectopic PTPRG-mCitrine expression, with a threshold of EGFR activation at around 6%–7% receptor occupancy with EGF (Figure 4B, top, blue line). The goodness of fit showed that this response was most consistent with a double-negative EGFR-PTPRG feedback. On the other hand, ectopic PTPRJ-mCitrine expression flattened the dose-response curve, revealing the underlying simple negative regulation (Figure 4B, bottom, blue line). Expression of the ER-bound PTPN2-mCitrine flattened the EGFR response, which could be equally well described by negative feedback or regulation (Figure 4B, middle, blue line). Fitting the dose response upon knockdown of PTPRJ with the three possible models (Figure S4A) revealed that the EGFR phosphorylation response could be best described with the double-negative feedback model (Figures 4A [bottom] and S4E; Table S1). This manifestation of the EGFR-PTPRG toggle switch indicates that the negative regulation by PTPRJ counters the switch-like EGFR phosphorylation response caused by PTPRG. The steepened dose response upon PTPN2 knockdown (Figure 4B, middle) indicates a negative regulation of autocatalytic EGFR phosphorylation by PTPN2. Consistent with siRNA-mediated knockdown, ectopic PTPRA-mCitrine expression did not affect the EGFR phosphorylation response (Figure S4D).

We investigated whether the biochemical mechanism behind the EGFR-PTPRG toggle switch originates from EGFR-induced activation of H_2O_2 production by NADPH oxidases (NOX) (Bae et al., 1997), which reversibly oxidizes the catalytic cysteine in PTPs to the catalytically impaired sulfenic acid (Salmeen et al., 2003). EGFR activation by EGF increased the production of H_2O_2 in MCF7 cells (Figures S4B and S4C). To first test whether the dose response of EGFR is indeed affected by H_2O_2, we inhibited NOX activity with diphenyleneiodonium (DPI). This converted the switch-like activation observed upon PTPRG-mCitrine expression to a gradual response (Figure 4C, top, yellow lines). Neither the EGF dose response upon ectopic expression of PTPN2-mCitrine nor PTPRJ-mCitrine or PTPRA-mCitrine was affected by DPI (Figures 4C [middle and bottom] and S4D). To then establish the connection between EGFR-induced H_2O_2 production and PTPRG inhibition, we determined whether the catalytic PTPRG cysteine is oxidized upon activation of EGFR by EGF. For this, cells were incubated for 10 min with dimedone, which reacts with the sulfenylated cysteine to form a stable thioether that is detectable by an anti-dimedone antibody (Seo and Carroll, 2009). The oxidation of the catalytic cysteine (Figures S4F and S4G) of PTPRG increased with EGF dose (Figures 4D [top] and S4G), confirming that the biochemical inhibitory link from EGFR to PTPRG in the toggle switch is generated by H_2O_2-mediated PTPRG inactivation. Neither PTPN2-mCitrine nor PTPRJ-mCitrine exhibited an EGF dose-dependent increase in catalytic cysteine oxidation (Figures 4D and S4G), consistent with the DPI experiments. To finally show that the EGF-induced oxidation of PTPRG occurs via EGFR-induced NOX activation, we knocked down the p22 subunit of NOX1-3 (p22^{Phox}), resulting in a strong reduction of EGF-induced PTPRG oxidation to levels observed following DPI inhibition (Figures 4E, S4H, and S4I).

These results therefore demonstrate that EGFR responsiveness to EGF is mainly determined by a double-negative feedback with PTPRG that is established by EGFR-mediated NOX-dependent production of H_2O_2 and modulated by PTPRJ activity at the plasma membrane and PTPN2 on the ER.

Dynamics of the Unified EGFR-PTP Network

To better understand how the EGFR-PTPRG toggle switch that determines sensitivity to EGF is modulated by negative regulation by PTPRJ and negative feedback by PTPN2, we transformed the spatial scheme that describes how vesicular dynamics enables PTPs to interact with EGFR (Figure 5A) into a unified causality diagram (Figure 5B). This enabled us to explore the dynamical properties of this network using 3D-bifurcation analysis (Strogatz, 2000). The phosphorylation dynamics of monomeric ligandless EGFR at the plasma membrane was analyzed theoretically as function of the system's parameters: liganded EGFR, and PTPRG/EGFR expression levels.

We first investigated the dynamical properties of the central EGFR-PTPRG double-negative motif (Figure 5B). Together with the activation of autocatalysis on ligandless EGFR by EGF-bound EGFR, this generates bistability for a large range of PTPRG/EGFR expression. This network motif thus determines at which EGF dose EGFR collectively activates (Figure 5C, green trajectories), but impedes signal shutdown since autocatalytic EGFR activation will persist after growth factor removal if the system is in the bistable region (Figure 5C, red trajectories). Lowering PTPRG expression (as with knockdown) pushes the system to the pre-activated state, as demonstrated in Figure 3F. This is alleviated by the negative regulation by PTPRJ, which narrows down the bistability region and shifts the system into the reversible bistable or even monostable region of the bifurcation diagram (Figure 5D, projected trajectories). The negative feedback with PTPN2 that is established by the vesicular recycling can play a role similar to that of PTPRJ. However, the vesicular recycling of activated EGFR monomers that are dephosphorylated by PTPN2 in the cytoplasm is also essential to maintain sufficient EGFR at the plasma membrane for autocatalysis to manifest.

Whether and how this EGFR-PTP system will respond to time-varying cues will depend on where the system is organized in parameter space (PTPRG/EGFR). To explore how the system will respond in the different parameter regimes, we simulated EGFR responsiveness to a train of EGF pulses. If the dynamics of the EGFR-PTP system is dominated by the bistable properties of the PTPRG-EGFR toggle switch, the simulation shows that EGFR will remain "trapped" in the active state after the first EGF pulse, thereby not being able to sense subsequent EGF cues (Figure 5E, left). However, if the system is organized close

(D) Quantification of PTPRG-mCitrine (top), PTPN2-mCitrine (middle), and PTPRJ-mCitrine (bottom) catalytic cysteine oxidation for different EGF-Alexa647 doses (blue bars, means ± SEM, N = 4–7; Figure S4G) and with 10 μM DPI pre-incubation (yellow bars, means ± SEM, N = 5; Figure S4G). p values given as numbers above the bars are calculated with respect to the unstimulated case.

(E) Quantification of PTPRG-mCitrine catalytic cysteine oxidation in control (left) and upon knockdown of NOX component p22^{phox} in MCF7 cells treated with 80 ng/mL EGF-Alexa647 with or without 10 μM DPI 20-min pre-incubation, or 4 mM H_2O_2 (mean ± SEM, N = 4, Figures S4H and S4I).

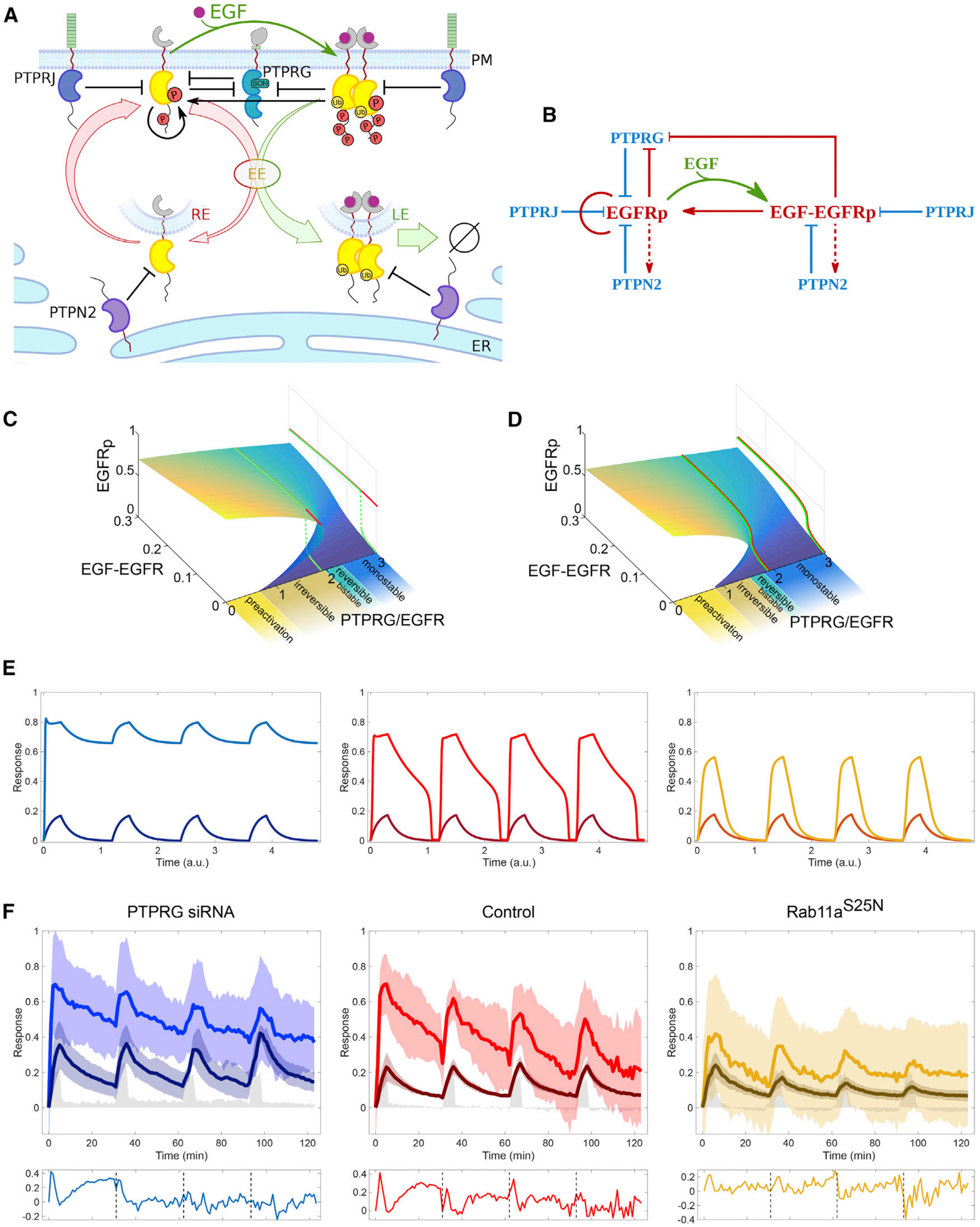

Figure 5. Dynamics of the Spatially Distributed EGFR-PTP Network

(A) Scheme of the EGFR-PTP interaction network established through EGFR trafficking dynamics. EGFR interacts with PTPRG/PTPRJ at the PM and PTPN2 in the cytoplasm. All notations as in Figure 1L.

(B) Causality diagram that corresponds to (A). Red/blue lines: causal interactions; green arrow: ligand binding.

(C) 3D-bifurcation diagram for double-negative EGFR-PTPRG feedback network topology at the plasma membrane, showing the dependence of monomeric EGFR phosphorylation (EGFRp) on PTPRG/EGFR expression ratio and fraction of liganded receptors. Forward (green) and backward (red) dose-response trajectories are shown for PTPRG/EGFR = 1.9, with corresponding orthographic projections on the right profile plane.

(D) 3D-bifurcation diagram as in (C), for the combined toggle-switch/negative regulation/negative-feedback network topology established by ligandless EGFR vesicular recycling. Projections are the same as in (C).

(E) Simulated temporal profiles of the fractions of liganded (dark) and phosphorylated receptors (light) in response to a train of pulses (gray), when the system is organized in the bistable regime (left), close to the bistability region (middle), and in the monostable regime (right) for the complete EGFR/PTP network as in (D).

(legend continued on next page)

to the bifurcation that denotes the transition to the bistable region, the response dynamics exhibit biphasic behavior with a rapid decay followed by slower relaxation (Figure 5E, middle). Further away from the bifurcation, in the monostable regime, EGFR phosphorylation closely follows the EGF input (Figure 5E, right).

To now identify where the EGFR-PTP system is poised and whether it can sense time-varying EGF signals, we administered four subsequent 5-min EGF-Alexa647 pulses followed by washout every 30 min to live MCF7 cells expressing EGFR-mTFP. The fraction of liganded receptor (EGF/EGFR = EGF − Alexa647/EGFR-mCitrine) as well as the fraction of phosphorylated EGFR (EGFRp = PTB-mCherry/EGFR-mCitrine) was ratiometrically determined at the plasma membrane as a function of time (STAR Methods). In control cells, EGFRp response relaxed in a biphasic way (with a fast and slow relaxation, light red lines in Figure 5F, middle) after each EGF pulse, reminiscent of the simulated response of a system poised close to the bifurcation. This differed from the relaxation of EGF/EGFR (Figure 5F, middle, lower box) that approximated a more monotonic decaying function (dark red lines), which is due to depletion of liganded receptors from the plasma membrane by endocytosis. The more rapid activation of EGFRp with respect to EGF/EGFR at the onset of each pulse is a clear manifestation of autocatalytic EGFR amplification (Figures 5F and 1D). This shows that the EGFR-PTP system has dynamical organization close to the bistable region, enabling both sensing and robust activation upon time-varying EGF stimuli.

PTPRG knockdown resulted in a response to EGF pulses within the limited boundaries of the upper activated state, which does not relax back to the basal inactivated state (light blue lines in Figure 5F, left). This is consistent with the persistent/bistable EGFR phosphorylation in the absence/low level of PTPRG (Figures 3E and 3F). This confirms that PTPRG is a central regulator of EGFR activation dynamics through a double-negative feedback motif. We also observed a subpopulation of cells (4 out of 9 cells) that relaxed back to the basal state after each EGF pulse resembling the control (Figure S4J), presumably due to variability in PTPRG knockdown with respect to EGFR expression levels. This reflects that PTPRG/EGFR concentration ratio dictates where on the bifurcation diagram the system is organized, thereby determining the dynamics of the system. Ectopic expression of dominant negative Rab11^{S25N} mutant impairs the vesicular recycling of EGFR monomers. This generates a lower steady-state abundance of EGFR at the plasma membrane, shifting the system to the monostable regime of the bifurcation diagram by effectively increasing the system parameter PTPRG/EGFR (Figure 5D). In this case, a dampened phosphorylation response to a train of EGF pulses was observed, whereby EGFRp follows closely the EGF/EGFR relaxation (Figure 5F, right, lower box). That recycling of EGFR monomers is essential to generate a sufficient concentration for autocatalytic amplification of phosphorylation at the plasma membrane after each EGF pulse is apparent from the strong decrease in both autocatalytic EGFR activation (Figure 5F, right) and the dampening of both EGFRp and EGF/EGFR after each pulse. In this case, the system loses its robustness in response to time-varying stimuli and becomes more rapidly insensitive to upcoming EGF pulses (Figure 5F, light orange lines). How long the system can respond to time-varying EGF stimuli generally depends on the total amount of expressed EGFR that is recycling in the cell, and how quickly this pool is depleted by the unidirectional trafficking of liganded EGFR, which in turn is determined by the magnitude of EGF stimuli.

DISCUSSION

By quantifying how cell-to-cell variability in PTP expression relates to EGFR phosphorylation, we obtained a measure of specific phosphatase activity in cells and thereby could identify receptor-like PTPRG/J at the plasma membrane and ER-bound PTPN2 as major dephosphorylating activities of EGFR. The reciprocal perturbation of siRNA-mediated PTP knockdown provided the additional information about their non-redundancy and physiological relevance. PTPN2 predominantly regulates the duration of EGFR signaling by dephosphorylating EGFR dimers as they unidirectionally traffic toward the perinuclear late endosome. On the other hand, the recursive interaction of PTPRG with autocatalytically activated monomeric receptors is the main determinant of EGFR's sensitivity to growth factor. This sensitivity is tightly modulated by the activity of PTPRJ at the plasma membrane, as well as that of PTPN2 during the constitutive recycling of monomeric receptors.

Endocytic traffic thus displaces EGFR dimers to the perinuclear cytoplasm to ensure finite signal duration, while vesicular recycling repopulates the plasma membrane with inactivated monomeric receptors (Baumdick et al., 2015) to maintain responsiveness to upcoming growth factor stimuli. The spatial segregation of high PTPN2 activity from the plasma membrane ensures that EGFR phosphorylation is not immediately suppressed upon exposure to ligand. This is also in line with the relatively low mRNA expression of the PTPRs with respect to PTPN2 (PTPR/ER-PTP mRNA $\sim$ 0.045, Figure S1A). The system therefore can initiate signaling due to a clear segregation of tyrosine kinase and phosphatase activity and shuts down by their colocalization over time due to vesicular traffic. This process extends signaling duration to tens of minutes, which cannot occur if signaling is regulated by the rapid diffusion-controlled recruitment of cytosolic phosphatases to the plasma membrane (Grecco et al., 2011). The other ER-bound PTPN1 (Frangioni et al., 1992) appeared as redundant with respect to PTPN2 in our CA-FLIM screen and likely performs a similar function, albeit acting later after EGF stimulus due to its more confined distribution in the perinuclear area (Figure 2C) (Baumdick et al., 2015; Haj et al., 2002; Yudushkin et al., 2007).

EGFR responsiveness to growth factors, on the other hand, is mainly determined by the dynamical features that emerge from

(F) Temporal traces of the fraction of ligand-bound (EGF-Alexa647/EGFR-mCitrine, dark color) and phosphorylated EGFR estimated by PTB-mCherry translocation to the plasma membrane (PTB-mCherry/EGFR-mCitrine, light color) in live MCF7 cells expressing non-targeting siRNA (middle, n = 4, N = 1), following siRNA-mediated knockdown of PTPRG (left, n = 5, N = 2), and ectopic Rab11^{S25N} expression (right, n = 16, N = 2). Data were acquired at 1-min intervals following 20 ng/mL 5P-EGF every 30 min. Means ± SD are shown. Lower boxes depict the normalized differences between the fraction of phosphorylated and liganded EGFR.

autocatalytic activation of monomeric EGFR (Reynolds et al., 2003; Tischer and Bastiaens, 2003) in concert with the EGFR-PTPRG toggle switch on the plasma membrane. The basis for autocatalytic EGFR activation is most likely the phosphorylation of the regulatory Y_{845} in the kinase activation loop, which stabilizes an active conformation of the receptor (Shan et al., 2012). This could be established by direct autophosphorylation or indirect phosphorylation by Src (Sato et al., 1995), which is in turn activated by EGFR (Osherov and Levitzki, 1994). Since ligand-bound EGFR can also initiate autocatalytic phosphorylation on ligandless receptors, EGFR phosphorylation can be rapidly amplified at low growth factor concentrations. This is opposed by the phosphatase activity of the tumor suppressor PTPRG (Kwok et al., 2015). However, NOX-dependent H_2O_2 production couples EGFR phosphorylation to the inactivation of PTPRG through oxidation of its catalytic cysteine, thus ensuring that rapid EGFR phosphorylation response takes place upon a threshold concentration of growth factor. The property of this bistable system to be irreversible after activation is opposed by the negative regulation through PTPRJ, which pushes the system toward reversible activation that is necessary to sense upcoming growth factor cues.

Growth factor receptors are the "sensory organs" of cells that perceive time-varying growth factor stimuli, leading to a variety of cellular responses. The dynamical organization of the EGFR-PTP system is poised such that EGFR signaling is only activated for physiological threshold concentrations of EGF and can robustly respond to time-varying stimuli in a non-stationary environment. Given the role of vesicular trafficking in the regulation of EGFR activation and signaling, changes in its vesicular dynamics may represent a mechanism through which environmental inputs such as cell-cell contact can influence the cellular response to EGF stimulation, generating contextual plasticity in growth factor signaling.

STAR★METHODS

Detailed methods are provided in the online version of this paper and include the following:

- KEY RESOURCES TABLE
- CONTACT FOR REAGENT AND RESOURCE SHARING
- EXPERIMENTAL MODEL AND SUBJECT DETAILS
  - Cell Culture
- METHOD DETAILS
  - Expression Plasmid Library
  - Antibodies
  - hEGF-Alexa647
  - PY72-Cy3.5 Labelling
  - Transfection and EGF Treatment
  - Reverse Transfection for CA-FLIM
  - Identifying the Optimal siRNA Concentration
  - In-Cell Westerns
  - Immunofluorescence
  - mRNA Profiling
  - Quantifying Ectopic EGFR-mTFP Expression in MCF7 Cells
  - Hydrogen Peroxide Measurements
  - Temporal H_2O_2 Profiles upon EGF Stimulation
  - Detection of PTP_X Catalytic Cysteine Oxidation
  - Anisotropy Microscopy
  - Fluorescence Lifetime Imaging Microscopy (FLIM)
  - Confocal Microscopy
  - Imaging EGFR Vesicular Dynamics
  - Multiple EGF Pulse Experiment
- QUANTIFICATION AND STATISTICAL ANALYSIS
  - Single Cell Segmentation and Quantification
  - Global Analysis of FLIM Data
  - CA-FLIM Identification of PTPs that Dephosphorylate EGFR
  - Relative Specific PTP_X -mCitrine Activity
  - Spatial-Temporal Maps (STMs)
  - Determining PTP_X Reactivity towards Phosphorylated EGFR
  - Ligandless EGFR Recycling Rates
  - Live Cell Dose Response Imaging and Quantification
  - Modeling EGFR Phosphorylation Dynamics
- DATA AND SOFTWARE AVAILABILITY

SUPPLEMENTAL INFORMATION

Supplemental Information includes four figures, three tables, and two videos and can be found with this article online at https://doi.org/10.1016/j.cels.2018.06.006.

ACKNOWLEDGMENTS

The project was partly funded by the European Research Council (ERC AdG 322637) and the European Commission (grant 278568) to P.I.H.B. P.R.-N. was supported by Marie Curie Actions (IEF 219743). The authors thank Kirsten Michel for help with biochemical experiments, Dr. Jan Hübinger for help with setting up the EGF-pulse instrumentation, and Drs. Astrid Krämer, Peter Bieling, and Malte Schmick for critically reading the manuscript.

AUTHOR CONTRIBUTIONS

P.I.H.B. conceived the project. P.I.H.B. and A.K. developed the dynamical system concepts and supervised the study. H.E.G. designed experiments and S.F., P.R.-N., H.E.G., K.C.S., and A.M. performed the FLIM screening experiments; A.M., A.S., K.C.S., W.S., and M.B. performed EGFR phosphorylation and trafficking experiments; M.B. performed anisotropy experiments; A.S., R.S., and Y.B. performed single-cell dose-response experiments; M.S.J., R.S., and A.M. performed oxidation assays; and A.S. and W.S. performed EGF-pulse experiments. L.R. and J.L. generated the PTP cDNA plasmid library. A.S., H.E.G., K.C.S., and A.K. performed image and data analysis. P.I.H.B., A.K., and A.S. developed the theoretical models. P.I.H.B. and A.K. wrote the manuscript with help of A.S. and A.M.

DECLARATION OF INTERESTS

The authors declare no competing interests.

Received: August 4, 2017
Revised: December 22, 2017
Accepted: June 7, 2018
Published: August 22, 2018

REFERENCES

Alonso, A., Sasin, J., Bottini, N., Friedberg, I., Friedberg, I., Osterman, A., Godzik, A., Hunter, T., Dixon, J., and Mustelin, T. (2004). Protein tyrosine phosphatases in the human genome. Cell *117*, 699–711.

Andersen, J.N., Mortensen, O.H., Peters, G.H., Drake, P.G., Iversen, L.F., Olsen, O.H., Peter, G., Andersen, H.S., Tonks, N.K., Møller, P.H., et al.

(2001). Structural and evolutionary relationships among protein tyrosine phosphatase domains. Mol. Cell. Biol. *21*, 7117–7136.

Arkhipov, A., Shan, Y., Das, R., Endres, N.F., Eastwood, M.P., Wemmer, D.E., Kuriyan, J., and Shaw, D.E. (2013). Architecture and membrane interactions of the EGF receptor. Cell *152*, 557–569.

Bae, Y.S., Kang, S.W., Seo, M.S., Baines, I.C., Tckle, E., Chock, P.B., and Rhee, S.G. (1997). Epidermal growth factor (EGF)-induced generation of hydrogen peroxide. Role in EGF receptor-mediated tyrosine phosphorylation. J. Biol. Chem. *272*, 217–221.

Barr, A.J., Ugochukwu, E., Lee, W.H., King, O.N.F., Filippakopoulos, P., Alfano, I., Savitsky, P., Burgess-Brown, N.A., Müller, S., and Knapp, S. (2009). Large-scale structural analysis of the classical human protein tyrosine phosphatome. Cell *136*, 352–363.

Baumdick, M., Brueggemann, Y., Schmick, M., Xouri, G., Sabet, O., Davis, L., Chin, J.W., and Bastiaens, P.I.H. (2015). EGF-dependent re-routing of vesicular recycling switches spontaneous phosphorylation suppression to EGFR signaling. Elife *4*, https://doi.org/10.7554/eLife.12223.

Belle, L., Ali, N., Lonic, A., Li, X., Paltridge, J.L., Roslan, S., Herrmann, D., Conway, J.R., Gehling, F.K., Bert, A.G., et al. (2015). The tyrosine phosphatase PTPN14-Pez inhibits metastasis by altering protein trafficking. Sci. Signal. *8*, ra18.

Berrow, N.S., Alderton, D., Sainsbury, S., Nettleship, J., Assenberg, R., Rahman, N., Stuart, D.I., and Owens, R.J. (2007). A versatile ligation-independent cloning method suitable for high-throughput expression screening applications. Nucleic Acids Res. *35*, e45.

Bilan, D.S., Pase, L., Joosen, L., Gorokhovatsky, A.Y., Ermakova, Y.G., Gadella, T.W.J., Grabher, C., Schultz, C., Lukyanov, S., and Belousov, V.V. (2013). HyPer-3: a genetically encoded H_2O_2 probe with improved performance for ratiometric and fluorescence lifetime imaging. ACS Chem. Biol. *8*, 535–542.

Charafe-Jauffret, E., Ginestier, C., Monville, F., Finetti, P., Adélaïde, J., Cervera, N., Fekairi, S., Xerri, L., Jacquemier, J., Birnbaum, D., et al. (2005). Gene expression profiling of breast cell lines identifies potential new basal markers. Oncogene *25*, 2273–2284.

Chib, S., and Greenberg, E. (1995). Understanding the Metropolis-Hastings algorithm. Am. Stat. *49*, 327.

Dickinson, B.C., Peltier, J., Stone, D., Schaffer, D.V., and Chang, C.J. (2011). Nox2 redox signaling maintains essential cell populations in the brain. Nat. Chem. Biol. *7*, 106–112.

Flint, A.J., Tiganis, T., Barford, D., and Tonks, N.K. (1997). Development of "substrate-trapping" mutants to identify physiological substrates of protein tyrosine phosphatases. Proc. Natl. Acad. Sci. USA *94*, 1680–1685.

Frangioni, J.V., Beahm, P.H., Shifrin, V., Jost, C.A., and Neel, B.G. (1992). The nontransmembrane tyrosine phosphatase PTP-1B localizes to the endoplasmic reticulum via its 35 amino acid C-terminal sequence. Cell *68*, 545–560.

Fueller, J., Egorov, M.V., Walther, K.A., Sabet, O., Mallah, J., Grabenbauer, M., and Kinkhabwala, A. (2015). Subcellular partitioning of protein tyrosine Phosphatase 1b to the endoplasmic reticulum and mitochondria depends sensitively on the composition of its tail anchor. PLoS One *10*, e0139429.

Glenney, J.R., Zokas, L., and Kamps, M.P. (1988). Monoclonal antibodies to phosphotyrosine. J. Immunol. Methods *109*, 277–285.

Grecco, H.E., Roda-Navarro, P., Girod, A., Hou, J., Frahm, T., Truxius, D.C., Pepperkok, R., Squire, A., and Bastiaens, P.I.H. (2010). In situ analysis of tyrosine phosphorylation networks by FLIM on cell arrays. Nat. Methods *7*, 467–472.

Grecco, H.E., Schmick, M., and Bastiaens, P.I.H. (2011). Signaling from the living plasma membrane. Cell *144*, 897–909.

Haj, F.G., Verveer, P.J., Squire, A., Neel, B.G., and Bastiaens, P.I.H. (2002). Imaging sites of receptor dephosphorylation by PTP1B on the surface of the endoplasmic reticulum. Science *295*, 1708–1711.

Hendriks, B.S., Opresko, L.K., Wiley, H.S., and Lauffenburger, D. (2003). Quantitative analysis of HER2-mediated effects on HER2 and epidermal growth factor receptor endocytosis. Distribution of homo- and heterodimers depends on relative HER2 levels. J. Biol. Chem. *278*, 23343–23351.

Hipel, K.W. (1981). Geophysical model discrimination using the Akaike information criterion. IEEE Trans. Automat. Contr. *26*, 358–378.

José, J., Noz, M., Arrega, C., Blanco-Aparicio, C., and Pulido, R. (2003). Differential interaction of the tyrosine phosphatases PTP-SL, STEP and HePTP with the mitogen-activated protein kinases ERK1/2 and p38α is determined by a kinase specificity sequence and influenced by reducing agents. Biochem. J. *372*, 193–201.

Kamentsky, L., Jones, T.R., Fraser, A., Bray, M.A., Logan, D.J., Madden, K.L., Ljosa, V., Rueden, C., Eliceiri, K.W., and Carpenter, A.E. (2011). Improved structure, function and compatibility for cellprofiler: modular high-throughput image analysis software. Bioinformatics *27*, 1179–1180.

Konitsiotis, A.D., Roßmannek, L., Stanoev, A., Schmick, M., and Bastiaens, P.I.H. (2017). Spatial cycles mediated by UNC119 solubilisation maintain Src family kinases plasma membrane localisation. Nat. Commun. *8*, 114.

Koseska, A., and Bastiaens, P.I. (2017). Cell signaling as a cognitive process. EMBO J. *36*, 568–582.

Kovacs, E., Das, R., Wang, Q., Collier, T.S., Cantor, A., Huang, Y., Wong, K., Mirza, A., Barros, T., Grob, P., et al. (2015). Analysis of the role of the C-terminal tail in the regulation of the epidermal growth factor receptor. Mol. Cell. Biol. *35*, 3083–3102.

Kwok, A., Cheung, L., Chok, J., Ip, Y., Chi, A., Chu, H., Man, M., Leong, L., Mun, J., Ko, Y., et al. (2015). PTPRG suppresses tumor growth and invasion via inhibition of Akt signaling in nasopharyngeal carcinoma. Oncotarget *6*, 13434–13447.

Lauffenburger, D.A., and Linderman, J.J. (1996). Receptors: Models for Binding, Trafficking, and Signaling (Oxford University Press).

Li, M.Z., and Elledge, S.J. (2007). Harnessing homologous recombination in vitro to generate recombinant DNA via SLIC. Nat. Methods *4*, 251–256.

Lim, W.A., and Pawson, T. (2010). Phosphotyrosine signaling: evolving a new cellular communication system. Cell *142*, 661–667.

Liu, F., and Chernoff, J. (1997). Protein tyrosine phosphatase 1B interacts with and is tyrosine phosphorylated by the epidermal growth factor receptor. Biochem. J. *327*, 139–145.

Lorenzen, J.A., Dadabay, C.Y., and Fischer, E.H. (1995). COOH-terminal sequence motifs target the T cell protein tyrosine phosphatase to the ER and nucleus. J. Cell Biol. *131*, 631–643.

Masip, M.E., Huebinger, J., Christmann, J., Sabet, O., Wehner, F., Konitsiotis, A., Fuhr, G.R., and Bastiaens, P.I.H. (2016). Reversible cryo-arrest for imaging molecules in living cells at high spatial resolution. Nat. Methods *13*, 665–672.

Meng, T.C., Fukada, T., and Tonks, N.K. (2002). Reversible oxidation and inactivation of protein tyrosine phosphatases in vivo. Mol. Cell *9*, 387–399.

van Munster, E.B., and Gadella, T.W., Jr. (2004). Suppression of photobleaching-induced artifacts in frequency-domain FLIM by permutation of the recording order. Cytometry A *58*, 185–194.

Offterdinger, M., Georget, V., Girod, A., and Bastiaens, P.I.H. (2004). Imaging phosphorylation dynamics of the epidermal growth factor receptor. J. Biol. Chem. *279*, 36972–36981.

Okutani, T., Okabayashi, Y., Kido, Y., Sugimoto, Y., Sakaguchi, K., Matuoka, K., Takenawa, T., and Kasuga, M. (1994). Grb2/Ash binds directly to tyrosines 1068 and 1086 and indirectly to tyrosine 1148 of activated human epidermal growth factor receptors in intact cells. J. Biol. Chem. *269*, 31310–31314.

Oliner, J.D., Kinzler, K.W., and Vogelstein, B. (1993). In vivo cloning of PCR products in *E. coli*. Nucleic Acids Res. *21*, 5192–5197.

Osherov, N., and Levitzki, A. (1994). Epidermal growth factor dependent activation of the Src family kinases. Eur. J. Biochem. *225*, 1047–1053.

Otsu, N. (1979). A threshold selection method from gray-level histograms. IEEE Trans. Syst. Man. Cybern. *9*, 62–66.

Roda-Navaro, P., and Bastiaens, P.I.H. (2014). Dynamic recruitment of protein tyrosine phosphatase PTPD1 to EGF stimulation sites potentiates EGFR activation. PLoS One *9*, e103203.

Reynolds, A.R., Tischer, C., Verveer, P.J., Rocks, O., and Bastiaens, P.I.H. (2003). EGFR activation coupled to inhibition of tyrosine phosphatases causes lateral signal propagation. Nat. Cell Biol. *5*, 447–453.

Salmeen, A., Andersen, J.N., Myers, M.P., Meng, T.-C., Hinks, J.A., Tonks, N.K., and Barford, D. (2003). Redox regulation of protein tyrosine phosphatase 1B involves a sulphenyl-amide intermediate. Nature *423*, 769–773.

Sato, K.I., Sato, A., Aoto, M., and Fukami, Y. (1995). c-SRC phosphorylates epidermal growth factor receptor on tyrosine 845. Biochem. Biophys. Res. Commun. *215*, 1078–1087.

Schlessinger, J. (2002). Ligand-induced, receptor-mediated dimerization and activation of EGF receptor. Cell *110*, 669–672.

Schmick, M., and Bastiaens, P.I.H. (2014). The interdependence of membrane shape and cellular signal processing. Cell *156*, 1132–1138.

Seo, Y.H., and Carroll, K.S. (2009). Profiling protein thiol oxidation in tumor cells using sulfenic acid-specific antibodies. Proc. Natl. Acad. Sci. USA *106*, 16163–16168.

Shan, Y., Eastwood, M.P., Zhang, X., Kim, E.T., Arkhipov, A., Dror, R.O., Jumper, J., Kuriyan, J., and Shaw, D.E. (2012). Oncogenic mutations counteract intrinsic disorder in the EGFR kinase and promote receptor dimerization. Cell *149*, 860–870.

Sonntag, M.H., Ibach, J., Nieto, L., Verveer, P.J., and Brunsveld, L. (2014). Site-specific protection and dual labeling of human epidermal growth factor (hEGF) for targeting, imaging, and cargo delivery. Chemistry *20*, 6019–6026.

Strogatz, S.H. (2000). Nonlinear Dynamics and Chaos: with Applications to Physics, Biology, Chemistry, and Engineering (Westview Press).

Tarcic, G., Boguslavsky, S.K., Wakim, J., Kiuchi, T., Liu, A., Reinitz, F., Nathanson, D., Takahashi, T., Mischel, P.S., Ng, T., et al. (2009). An unbiased screen identifies DEP-1 tumor suppressor as a phosphatase controlling EGFR endocytosis. Curr. Biol. *19*, 1788–1798.

Tiganis, T., Bennett, A.M., Ravichandran, K.S., and Tonks, N.K. (1998). Epidermal growth factor receptor and the apaptor protein p52Shc are specific substrates of T-cell protein tyrosine phoshphatase. Mol. Cell. Biol. *18*, 1622–1634.

Tischer, C., and Bastiaens, P.I.H. (2003). Opinion: lateral phosphorylation propagation: an aspect of feedback signalling? Nat. Rev. Mol. Cell Biol. *4*, 971–975.

Tonks, N.K. (2006). Protein tyrosine phosphatases: from genes, to function, to disease. Nat. Rev. Mol. Cell Biol. *7*, 833–846.

Wagner, M.J., Stacey, M.M., Liu, B.A., and Pawson, T. (2013). Molecular mechanisms of SH2- and PTB-domain-containing proteins in receptor tyrosine kinase signaling. Cold Spring Harb. Perspect. Biol. *5*, 1–20.

Wouters, F.S., and Bastiaens, P.I.H. (1999). Fluorescence lifetime imaging of receptor tyrosine kinase activity in cells. Curr. Biol. *9*, 1127–1130.

Yao, Z., Darowski, K., St-Denis, N., Wong, V., Offensperger, F., Villedieu, A., Amin, S., Malty, R., Aoki, H., Guo, H., et al. (2017). A global analysis of the receptor tyrosine kinase-protein phosphatase interactome. Mol. Cell *65*, 347–360.

Yarden, Y., and Sliwkowski, M.X. (2001). Untangling the ErbB signalling network. Nat. Rev. Mol. Cell Biol. *2*, 127–137.

Yuan, T., Wang, Y., Zhao, Z.J., and Gu, H. (2010). Protein-tyrosine phosphatase PTPN9 negatively regulates ErbB2 and epidermal growth factor receptor signaling in breast cancer cells. J. Biol. Chem. *285*, 14861–14870.

Yudushkin, I.A., Schleifenbaum, A., Kinkhabwal, A., Neel, B.G., Schultz, C., and Bastiaens, P.I.H. (2007). Live-cell imaging of enzyme-substrate interaction reveals spatial regulation of PTP1B. Science *315*, 115–119.

STAR★METHODS

KEY RESOURCES TABLE

REAGENT or RESOURCE	SOURCE	IDENTIFIER
Antibodies		
Mouse monoclonal antibody PY72	InVivo Biotech Services	P172.1
Rabbit anti EGFR pY_{1045}	Cell Signaling Technology	Cat. # 2237
Rabbit anti EGFR pY_{1068}	Cell Signaling Technology	Cat. # 3777
Mouse anti EGFR pY_{845}	BD Biosciences	Cat. # 558381
Goat anti EGFR	R&D Systems	Cat. # AF231
Alexa Fluor® 568 donkey anti-rabbit IgG	Life Technologies	Cat. # A10042
Alexa Fluor® 568 donkey anti-mouse IgG	Life Technologies	Cat. # A10037
Alexa Fluor® 647 donkey anti-goat IgG	Life Technologies	Cat. # A-21447
Alexa Fluor® 647 chicken anti-mouse IgG	Life Technologies	Cat. # A-21463
Alexa Fluor® 647 donkey anti-rabbit IgG	Life Technologies	Cat. # A-31573
IRDye® 800CW Donkey anti-Rabbit IgG	Licor	Cat. # 926-32213
IRDye® 680RD Donkey anti-Mouse IgG	Licor	Cat. # 926-68072
Goat anti GFP	Abcam	Cat. #ab6673
Anti-GFP antibody	Clontech	Cat. #632375
Anti-Sulfenic acid modified cysteine antibody	Millipore	Cat. #ABS30
Anti-EGFR antibody	Cell Signaling Technology	Cat. #4267
Bacterial and Virus Strains		
E. coli (XL10 - Gold ultracompetent) cells	Stratagene	Cat. # 200314
Chemicals, Peptides, and Recombinant Proteins		
Dulbecco's modified Eagle's medium (DMEM)	PAN Biotech	Cat. # P04-01500
Dulbecco's modified Eagle's medium (DMEM / F12)	PAN Biotech	Cat. # P04-41450
MEM Amino Acids Solution (50x)	PAN Biotech	Cat. # P08 32100
Penicillin- Streptomycin	PAN Biotech	Cat. # P06 07100
Fetal Bovine Serum	Sigma-Aldrich	Cat. # F7524
EGF	Sigma-Aldrich	Cat. # E9644
Hydrocortisone	Sigma-Aldrich	Cat. #H-0888
Cholera toxin	Sigma-Aldrich	Cat. #C-8052
Insulin	Sigma-Aldrich	Cat. #I-1882
Cy3.5® NHS ester	GE Healthcare	Cat. # PA23501
FuGENE6	Roche Diagnostics	Cat. # 11 815 091
Roti® Histofix	Carl Roth	Cat. # P087
Odyssey Blocking buffer	LI-COR	Cat. # 927
RNA Later	Thermo Fisher Scientific	Cat. # AM7020
PF6-AM	Dickinson et al., 2011	Prof Christopher J. Chang, University of California, Berkeley
N-Ethylmaleimide	Sigma-Aldrich	Cat. # 128-53-0
Dimedone	Sigma-Aldrich	Cat. # 38490
Phusion Flash High-Fidelity PCR Master Mix	Thermo Fisher Scientific	Cat. # F548S
Herculase II Fusion DNA Polymerase	Agilent	Cat. # 600675
Triton-X100	Serva	Cat. # 37240
Alexa647-Malemide	Thermo Fisher Scientific	Cat. # A20347
N,N dimethylformamide	Acros Organics	Cat. # 348435000
Bicine	Sigma-Aldrich	Cat. # B3876
Diphenyleneiodonium	Sigma-Aldrich	Cat. # D2926

(*Continued on next page*)

Continued		
REAGENT or RESOURCE	SOURCE	IDENTIFIER
Critical Commercial Assays		
MycoAlert Mycoplasma detection kit	Lonza	Cat. # LT07-218
RNeasy Maxi Kit	QIAGEN	Cat. # 75162
Oligotex mRNA Midi Kit	QIAGEN	Cat. # 70042
AffinityScript Multiple Temperature cDNA Synthesis Kit	Agilent	Cat. #200436
BigDye® Terminator v3.1 Cycle Sequencing Kit	Thermo Fisher Scientific	Cat. # 4337455
PureYield plasmid Midiprep System	Promega	Cat. # A2492
NucleoBond® Xtra Midi EF	Macherey-Nagel	Cat. # 740420.10
Experimental Models: Cell Lines		
MCF-7	ECACC	Cat. No. 86012803
MCF7-EG	Roda-Navaro and Bastiaens, 2014	N/A
MCF10A	ATCC	CRL - 10317
Oligonucleotides		
PTP_X and CYBA siRNA SMARTpool	Dharmacon	Additional Data Table S3 (separate file)
Recombinant DNA		
cHyPer3	Bilan et al., 2013	Prof. Vsevolod Belousov, Shemyakin–Ovchinnikov Institute of bioorganic chemistry, Moscow
His-CBD-Intein-(Cys)-hEGF-(Cys)	Sonntag et al., 2014	Prof. Luc Brunsveld, University of Technology, Eindhoven
cDNA-mCitrine expression plasmid constructs	This paper	Additional Data Table S2 (separate file)
EGFR-mCitrine-N1	Baumdick et al., 2015	N/A
PTB-mCherry	Fueller et al., 2015	N/A
cCbl-BFP	Fueller et al., 2015	N/A
Rab11-S25N-BFP	Konitsiotis et al., 2017	N/A
Software and Algorithms		
CellProfiler	Kamentsky et al., 2011	http://cellprofiler.org
R Statistical Software	Foundation for Statistical Computing	https://www.r-project.org/
XPPAUT		www.math.pitt.edu/~bard/xpp/xpp.html)
MATLAB	MathWorks	http://mathworks.com/
Python	Python Software Foundation	https://www.python.org/
Scikit-image	Version 0.11.3	www.scikit-image.org
OpenCV	Version 2.4.8	http://opencv.org
ImageJ/Fiji		https://fiji.sc/
IgorPro 6.37	Wavemetrics	www.igorpro.net
Other		
7K Zeba Spin Desalting Columns	Thermo Scientific	Cat. # 89882
Dynabeads® Protein G magnetic beads	Thermo Fisher Scientific	Cat. # 10003D
NuPAGE Novex 4-12% Bis-Tris gels	ThermoFisher	Cat. # NP0335BOX
Cellasic ONIX plates	Merck Chemicals	M04S

CONTACT FOR REAGENT AND RESOURCE SHARING

Further information and requests for resources and reagents should be directed to and will be fulfilled by the Lead Contact, Prof. Dr. Philippe I. H. Bastiaens (philippe.bastiaens@mpi-dortmund.mpg.de)

EXPERIMENTAL MODEL AND SUBJECT DETAILS

Cell Culture

MCF-7 cells (sex: female, ECACC, Cat. No. 86012803) and MCF7 cells stably expressing EGFR-EGFP ($EGFR^{-EGFP}$ MCF7) were cultured in Dulbecco's modified Eagle's medium (DMEM) (PAN Biotech), supplemented with 10% heat-inactivated fetal bovine

serum (FBS) (PAN Biotech), 10mM glutamine (PAN Biotech) and 1% Non-Essential Amino Acids (PAN Biotech) at 37°C with 5% CO_2. MCF10A (sex: female, ATCC-CRL 10317) were grown in DMEM/F12 media supplemented with 5% horse serum, 20ng/ml EGF (Sigma-Aldrich), 0.5μg/ml hydrocortisone (Sigma #H-0888), 100ng/ml cholera toxin (Sigma), 10μg/ml insulin (Sigma) and 1% glutamine. MCF7 and MCF10A cells were authenticated by Short Tandem Repeat (STR) analysis and did not contain DNA sequences from mouse, rat and hamster (Leibniz-Institut DSMZ). Cells were regularly tested for mycoplasma contamination using MycoAlert Mycoplasma detection kit (Lonza).

METHOD DETAILS

Expression Plasmid Library

The p2297-OPIN(n)mCitrine (Berrow et al., 2007) and p2150-OPIN(c)mCitrine (Berrow et al., 2007) vectors without His6-Tag were used to generate N- or C-terminally tagged PTP_X–mCitrine expression constructs. See Table S2 for PTP_X constructs with mRNA reference ID, source of the cDNA/ORF, vector, sequence of the Ligation-Independent-Cloning-(LIC) primers and any sequence discrepancies. To obtain PTP ORFs from human cell lines, mRNA was isolated with the RNeasy Maxi and Oligotex mRNA Midi Kit (QIAGEN) followed by cDNA synthesis using the AffinityScript Multiple Temperature cDNA Synthesis Kit (Agilent). The cloning of ORF into the pOPIN vector was done with a combination of '*in vivo* cloning' (Oliner et al., 1993) and "sequence and ligase independent cloning (SLIC)" (Li and Elledge, 2007) by the Dortmund Protein Facility. The PCR reaction comprised of LIC primers and Phusion Flash High-Fidelity PCR Master Mix (Thermo Fisher Scientific) or Herculase II Fusion DNA Polymerase (Agilent). PTP_x-pOPIN sequences were validated using BigDye® Terminator v3.1 Cycle Sequencing Kit (Thermo Scientific). Plasmids were extracted from transformed E.coli XL - 10 Gold ultracompetent cells using a high content PureYield plasmid Midiprep System (Promega) and NucleoBond® Xtra Midi EF (Macherey-Nagel). Trapping mutants were generated for PTPs listed in Figure 2B. See Table S2 for site of mutation and the respective LIC and mutagenesis primer pairs. Mutations were introduced into the WT PTP_X cDNA by an overlap extension PCR and later cloned into the respective vector using LIC. EGFR-mTFP-N1, was generated from EGFR-mCitrine-N1 (Baumdick et al., 2015) using AgeI and NotI restriction enzymes to exchange mCitrine with mTFP1. The EGFR-QG-mCitrine construct has been previously described (Baumdick et al., 2015). The constructs of PTB-mCherry, EGFR-mCherry and cCbl-BFP were described previously (Baumdick et al., 2015; Fueller et al., 2015). cHyPer3 (Bilan et al., 2013) plasmid was kindly provided by Prof. Vsevolod Belousov, Shemyakin–Ovchinnikov Institute of Bioorganic Chemistry, Moscow.

Antibodies

Primary antibodies: Mouse monoclonal antibody PY72 (Glenney et al., 1988) (InVivo Biotech Services, Henningsdorf, Germany), rabbit anti EGFR pY_{1068} (Cell Signaling; 1:400), goat anti EGFR (R&D Systems; 1:300). Secondary antibodies: Alexa Fluor® 568 donkey anti-rabbit IgG (Life Technologies, 1:200), Alexa Fluor® 568 donkey anti-mouse IgG (Life Technologies, 1:200), Alexa Fluor® 647 donkey anti-goat IgG (Life Technologies, 1:200), Alexa Fluor® 647 chicken anti-mouse IgG (Life Technologies, 1:200), Alexa Fluor® 647 donkey anti-rabbit IgG (Life Technologies, 1:200), IRDye® 800CW Donkey anti-Rabbit IgG (Licor, 1:10000), IRDye® 680RD Donkey anti-Mouse IgG (Licor, 1:10000).

hEGF-Alexa647

The His-CBD-Intein-(Cys)-hEGF-(Cys) plasmid (Sonntag et al., 2014) was kindly provided by Prof. Luc Brunsveld, University of Technology, Eindhoven. Human EGF was purified from E. coli BL21 (DE3) and N-terminally labeled with Alexa647-maleimide as described previously (Sonntag et al., 2014) and stored in PBS at -20°C.

PY72-Cy3.5 Labelling

Cy3.5® NHS ester (GE Healthcare) was dissolved in 10μl of dried N,N dimethylformamide (SERVA Electrophoresis). For each reaction, 15μl of 1M Bicine (pH 9.0) and a 10-fold molar excess (to PY72) of Cy3.5 were added to 100μl PY72 (0.25mg/ml) in PBS. After 20min in the dark the reaction was terminated by adding 6μl of 0.2M Tris buffer (pH 6.8). Free dye was removed by using 7K Zeba Spin Desalting Columns (Thermo Scientific). The absorption (A) of the filtrate was measured at 280nm (PY72) and 581nm (Cy3.5). For immunostaining, labelled antibody (30μg/ml in PBS) with dye to protein ratio of 3 - 5 was used. $\left(\frac{Dye}{Protein} = \frac{A581*1.7}{(A280-0.24*A581)*1.5}\right)$

Transfection and EGF Treatment

$3x10^4$ MCF7 cells were seeded per well in an 8-well Lab-Tek chamber (Nunc). After 7-8h of seeding, cells were transfected with 0.125μg of each plasmid (EGFR-mTFP, PTP_X-mCitrine and cCBL-BFP) using FUGENE6 (Roche Diagnostics) and incubated overnight. Before EGF stimulation, cells were serum starved with supplemented DMEM (see above) for 6h. The cells were stimulated with a sustained or a 5min-pulse of 200ng/ml EGF-Alexa647. Cells were chemically fixed with Roti® Histofix 4% (Carl Roth) for 20min, washed three times with PBS and then permeabilized with 0.1% Triton-X/PBS (SERVA Electrophoresis) for 15min. Cells were stored in PBS at 4°C before immunostaining. For live cell EGFR trafficking experiments, MCF7 cells were seeded at ~$1.5x10^4$ cells/well in an 8-well Lab-Tek chamber (S-EGF, 200ng/ml) or ~$1.5x10^5$ cells/well in a 6-well dish with a cover slide (Masip et al., 2016) (5P-EGF, 200ng/ml) and transfected after 24h with a total of 0.22μg (8-well) or 1μg (6-well) of EGFR-mCitrine,

PTB-mCherry and cCbl-BFP expression plasmids. In experiments requiring siRNA transfection, cells were transfected 6h before cDNA transfection with DharmaFECT1 (Dharmacon) according to the manufacturer's instructions. Before EGF stimulation, cells were serum starved with supplemented DMEM for at least 6h. For live cell dose response experiments, MCF7 cells were seeded at ~2x10^4 cells/well in an 8-well Lab-Tek chamber and transfected after 24h using FUGENE6 (Roche Diagnostics) with 0.15μg TagBFP, EGFR-mTFP/EGFR-mCitrine, PTB-mCherry, PTPRG-/PTPRJ-/PTPRA-/PTPN2-mCitrine or Rab11^{S25N}-mTFP expression plasmids (where applicable) per well. Before EGF stimulation, cells were serum starved with supplemented DMEM with 0.5% FCS for at least 6h. For a subset of the dose-response experiments, H_2O_2 production was inhibited with 10 μM Diphenyleneiodonium (DPI) for 30min prior to stimulation and imaging. For live cell dose response anisotropy experiments, 1.5x10^5 MCF7 cells were seeded in a MatTek (MatTek Corporation) dish and transfected with 1.6μg EGFR-QG-mCitrine expression plasmid using FUGENE6 (Roche Diagnostics) after 24h. For the time-lapse anisotropy experiment with 5P-EGF or S-EGF stimulation, 1.5x10^5 MCF7 cells were seeded in a MatTek (MatTek Corporation) dish and transfected after 24h with 1.6μg EGFR-QG-mCitrine and 1μg cCbl-BFP expression plasmids using FUGENE6 (Roche Diagnostics). Before EGF stimulation, cells were serum starved with supplemented DMEM with 0.5% FCS for 6h.

Reverse Transfection for CA-FLIM

siRNA and cDNA arrays were prepared and stored as described previously (Grecco et al., 2010). Each array constituted of 384 siRNA or cDNA reverse transfection spots printed on a NaOH treated glass slide of 1-well Lab-Tek chamber (Nunc). Along with other components (Grecco et al., 2010), the transfection-spotting mixture comprised of either 0.67μM siRNA Smart-Pools (Dharmacon, Table S3) for the siRNA array or 0.5μg of EGFR-mTFP and PTP_X-mCitrine plasmid for the cDNA array. For siRNA arrays 2.5x10^5 MCF7 cells stably expressing EGFR-EGFP and for cDNA arrays 3x10^5 MCF7 cells were seeded and incubated for 48h. Before EGF stimulation, cells were serum starved with supplemented DMEM (see above) without FCS for 6h. The cells were stimulated for 5, 30 or 120min with a sustained or 5min-pulse of 200ng/ml EGF-Alexa647. Cells were fixed chemically with Roti® Histofix 4% (Carl Roth) for 20min, washed three times with PBS and then permeabilized with 0.1% Triton-X/PBS (SERVA Electrophoresis) for 15min. Cells were stored in PBS at 4°C before immunostaining.

Identifying the Optimal siRNA Concentration

2×10^5 of MCF7 cells were seeded in each well of a 6-well tissue culture dish and transfected after 24h using 50nM or 100nM siRNA specific for PTPN2, PTPRG, PTPRJ, PTPRA, DUSP3, CYBA or non-targeting control siRNA with Dharmafect1 according to the manufacturer's instructions. RNA was isolated 24h after transfection using the Quick-RNA MicroPrep kit (Zymo Research, Freiburg, Germany). For quantification of mRNA expression levels of interest, 1μg input RNA was used for reverse transcription using the High Capacity Reverse Transcription kit (Applied Biosystems) according to the manufacturer instructions. Commercially available TaqMan assays (Thermo Fisher), PTPN2(Hs00959888_g1), PTPRG(Hs00892788_m1), PTPRJ(Hs01119326_m1), PTPRA(Hs00160751_m1), DUSP3(Hs01115776_m1), GAPDH(Hs02786624_g1), CYBA(Hs00609145_m1) were used to detect the amplicons after each cycle of a qPCR reaction ran in an IQ5 real-time PCR system cycler (Bio-Rad). Cycling condition were as follows: 40 cycles of 95°C for 10s and 57°C for 30s. Data were analysed using the ΔΔCt method for determination of relative gene expression by normalisation to an internal control gene (GAPDH), and fold expression change was determined compared to the control siRNA sample. N=2-3 independent experiments were performed.

In-Cell Westerns

MCF7 and MCF10A cells were seeded on black, transparent bottomed 96-well plates (3340, Corning, Hagen, Germany) coated with poly-L-lysine (P6282, Sigma Aldrich), transfected 24h later when required and starved for 18h in DMEM containing 0.5% FCS prior to stimulation. After stimulation, cells were fixed with Roti-Histofix 4% (Carl Roth, Karlsruhe, Germany) for 5min at 37°C and permeabilized with 0.1% Triton X-100 (v/v) for 5min at room temperature. Samples were incubated in Odyssey TBS blocking buffer (LI-COR Biosciences, Lincoln, NE, USA) for 30min at room temperature. Primary antibodies were incubated overnight at 4°C and secondary antibodies (IRDyes, LI-COR Biosciences) were incubated in the dark for 1h at room temperature. All wash steps were performed with TBS (pH 7.4). Intensity measurements were made using the Odyssey Infrared Imaging System (LI-COR Biosciences). Quantification of the integrated intensity in each well was performed using the MicroArray Profile plugin (OptiNav Inc., Bellevue, WA, USA) for ImageJ v1.47 (http://rsbweb.nih.gov/ij/). Two to four technical replicates per conditions were obtained per experiment, and all data presented represents means ± s.e.m. from at least three independent biological experiments.

Immunofluorescence

Fixed and permeabilized cells were incubated with 200μl of Odyssey Blocking buffer (LI-COR) for 30min. Primary antibodies were applied for 1h and fluorescently tagged (Alexa568) secondary antibodies for 30min, all antibodies were diluted in Odyssey Blocking buffer (LI-COR). Cells were washed three times with PBS between each antibody incubation step. Cells were imaged in PBS at 37°C. N=18-20 independent biological experiments were performed for the different conditions presented in Figures 1A, 3A–3D, and S3E–S3G.

mRNA Profiling

MCF7 cells were trypsinized and 6x10^5 cells were suspended in 4ml RNAse free water (Thermo Scientific) with 1ml RNAlater (Thermo Scientific). mRNA extraction and profiling was performed by Comprehensive Biomarker Center GmbH, Heidelberg on an array designed by Agilent 60-mer Sure print technology. The mRNA levels were obtained from three independent runs.

Quantifying Ectopic EGFR-mTFP Expression in MCF7 Cells

MCF7 and MCF10A cells were seeded at ~3x10^4 per well in 8-well Lab-Tek chambers (Nunc). MCF7 cells were transfected with EGFR-mTFP as described previously (see Cell culture and transfection). After serum starvation for 6h, cells were washed once with PBS and treated with EGF-Alexa647 (100ng/ml) for 5min at 37°C. After stimulation, cells were fixed with Roti® Histofix 4% for 10min and their nuclei stained with Hoechst (1μg/ml in TBS) for 5min. Cells were imaged in TBS on a Leica TCS SP8 confocal microscope. The mean EGFR-mTFP and EGF-Alexa647 fluorescence intensity per cell was obtained after cell segmentation in CellProfiler (Kamentsky et al., 2011) for which the fluorescence of the nuclear stain (Hoechst) and the EGF-Alexa647 were used. The histograms (Kernel density distribution) obtained from single-cell mean EGF-Alexa647 intensities from three independent experiments are shown in Figure 1A.

Hydrogen Peroxide Measurements

Intracellular H_2O_2 levels were determined by PF6-AM (Dickinson et al., 2011) (kindly provided by Prof Christopher J. Chang, University of California, Berkeley) fluorescence. MCF7 cells were seeded on 4-well Lab-Tek dishes. The next day, cells were transfected with EGFR-mCherry expression plasmid as described in Transfection above. After starvation in DMEM containing 0.5% FCS for 5-6h, cells were loaded with 5μM PF6-AM in DMEM for 30min at 37°C with or without 320ng/ml EGF-Alexa647. For NOX inhibition, cells were incubated with 10μM DPI 20min before PF6-AM loading. The cells were then washed twice with fresh DMEM and imaged immediately in DMEM (with 25mM HEPES, without Phenol Red) on a Leica TCS SP8 confocal microscope.

Temporal H_2O_2 Profiles upon EGF Stimulation

MCF7 cells were transfected with EGFR, cHyPer3 and C1-mCherry (Clontech) expression plasmids as described previously (see Cell culture and transfection). Cells were starved in DMEM containing 0.5% FCS for 5-6h and the medium was exchanged to Hank's Balanced Salt Solution (HBSS) supplemented with 20mM HEPES, pH=7.4. Images were acquired at 1min interval for 20mins on a Leica TCS SP8 confocal microscope. EGF-Alexa647 was added at 5min to a final concentration of 320ng/ml. N=2 independent experiments were performed.

Detection of PTP_X Catalytic Cysteine Oxidation

MCF7 cells were seeded at ~3×10^5 cells/well in a 6-well culture dish (Nunc) and transfected with 1μg PTP_X-mCitrine and 1μg EGFR expression plasmids per well. Prior stimulation cells were starved for 6h in supplemented DMEM with 0.5% FCS, followed by treatment with 25mM Dimedone (Sigma-Aldrich) for 10min at 37°C together with EGF-Alexa647 or H_2O_2 in serum-free medium. For NOX inhibition, cells were incubated with 10μM DPI for 20min at 37°C prior to Dimedone treatment. After incubation, cells were washed in ice-cold PBS supplemented with 100mM N-Ethylmaleimide (NEM, Sigma-Aldrich) and lysed in 85μL ice-cold lysis buffer (50mM Tris-HCl, pH 7.9, 150mM NaCl, 1% IGEPAL, 0.5% Na deoxycholate, 20mM NEM and protease inhibitors). For immunoprecipitation, equal amounts of protein lysates were incubated with Dynabeads® Protein G magnetic beads (ThermoFisher) and subsequently incubated with anti-GFP antibody overnight at 4°C. Lysates were incubated for 2h with Dynabeads® Protein G for pull down. Total and immunoprecipitated (IP) proteins were resolved by SDS/PAGE using NuPAGE Novex 4-12% Bis-Tris gels (ThermoFisher) in MOPS running buffer, transferred to PVDF membrane and then blocked with LI-COR blocking buffer (LI-COR Biosciences) for 1h. The membrane was then incubated with Anti-Sulfenic acid modified cysteine antibody (Seo and Carroll, 2009) and anti-GFP antibody overnight at 4°C. Next, the membrane was washed with TBS/T and incubated with the respective secondary antibodies for 1h. After washing with TBS/T, the blot was scanned using an Odyssey Infrared Imaging System (LI-COR). Western blot (WB) images were analyzed using FIJI (https://fiji.sc/) and Igor Pro 6.37 (http://www.igorpro.net/). For the temporal cysteine oxidation profiles, MCF-7 cells were stimulated with 5P-EGF in supplemented DMEM. Cells were incubated with 25mM Dimedone 10min before stopping the reaction by ice-cold PBS. N=4-7 independent experiments were performed per PTP.

Anisotropy Microscopy

Anisotropy microscopy was performed on an Olympus IX81 inverted microscope (Olympus Life Science) equipped with a MT20 illumination system and a temperature controlled CO_2 incubation chamber at 37°C and 5% CO_2. A linear dichroic polarizer (Meadowlark Optics) was implemented in the illumination path of the microscope and two identical polarizers were placed in an external filter wheel at orientations parallel and perpendicular to the polarization of the excitation light. Fluorescence images were collected via a 20x/0.75 NA air objective using an Orca CCD camera (Hamamatsu Photonics). BFP fluorescence emission was detected between 420-460 nm, mCitrine fluorescence emission between 495-540 nm and Alexa647 fluorescence emission between 705-745 nm.

For each field of view two images were acquired in the mCitrine channel, one with the emission polarizer oriented parallel to the excitation polarizer ($I_{\parallel}$) and one with the emission polarizer oriented perpendicular to the excitation polarizer ($I_{\perp}$). Fluorescence anisotropy (r^i) was calculated in each pixel i by:

$$r^i = \frac{G_i I_{\parallel} - I_{\perp}}{G_i I_{\parallel} + 2I_{\perp}}$$

The G-factor (G_i) was determined by acquiring the ratio of the parallel and perpendicular intensities of Fluorescein in a solution with a steady-state anisotropy close to zero. The CellR software supplied by the microscope manufacturer controlled data acquisition. Live cells were imaged in vitamin-free media in MatTeks and stimulated with EGF-Alexa647. Images were background-substracted and masks of the plasma membrane of single cells were generated from the EGFR-QG-mCitrine images using FIJI (https://fiji.sc/). N=3 independent experiments were performed for the dose-response as well as the temporal experiments shown in Figures 1 and S1.

Fluorescence Lifetime Imaging Microscopy (FLIM)

Cell arrays were imaged by automated microscopy as described previously (Grecco et al., 2010). An Olympus IX81 microscope (Olympus Life Science) was adapted for frequency domain FLIM. Samples were excited by an Argon laser (Coherent Innova 300C), externally modulated at 79.2MHz through an acousto-optic modulator (AOM, Intra Action SWM-804AE1-1) and fluorescence emission was recorded by a modulated intensified CCD camera (LaVision PicoStar HR / LaVision Imager QE). Both, AOM and image intensifier were modulated with coupled frequency generators (National Instruments PXI-5404). Image stacks were recorded in permuted phase order to reduce bleaching artefacts in the calculation of phase and modulation (van Munster and Gadella, 2004). The setup was controlled by a program developed in-house using LabVIEW 2010 (National Instruments). Phase and modulation were calibrated with a reflective aluminum foil located at the sample plane and drift-corrected with a mirror mounted in a filter cube.

Each cell array microscopy experiment comprised four arrays (for the four different time points: 0, 5, 30 and 120min) glued to a sample holder. The coordinates of the transfection spots were calibrated by automatic localization of six inked reference spots in transmission microscopy with a low magnification objective (UPlanSApo 4x/0.16 NA). To optimize the recording of the number of cells per spot, the array was pre-scanned in the mCitrine channel with a UPlanApo 10x/0.4 NA objective. The screening then proceeded in two runs with a UPlanSApo 40x/0.9 NA objective, first to obtain the donor-only fluorescence lifetime, followed by a second run after a 4h incubation period with PY72-Cy3.5 to obtain the FRET-FLIM dataset. N=6 independent experiments were performed. n=30-40 cells per experiment per condition were obtained.

The high-content FLIM screening experiments were performed similarly, but the positions were not selected automatically. Here, 2-4 positions in each well were defined and 16-25 fields of view around the selected coordinates were scanned to obtain data from a large number of cells. The complex Fourier components were computed from the phase stack using singular value decomposition. All the data acquired for the same donor molecule (EGFR-EGFP or EGFR-mTFP) and the same batch of labelled antibody (PY72-Cy3.5) was pooled together and jointly analyzed by global analysis (Grecco et al., 2010).

Confocal FLIM experiments to measure EGFR-PTP interactions were performed using a time-correlated single-photon counting module (LSM Upgrade Kit, PicoQuant) on an Olympus FV1000 confocal microscope (see: Confocal microscopy). Pulsed lasers were controlled with the Sepia II software (PicoQuant) at a pulse repetition frequency of 40MHz. The sample was excited using a 440nm diode laser (LDH 440, PicoQuant). Fluorescence emission was spectrally filtered using a narrow-band emission filter (HQ 480/20, Chroma). Photons were detected using a single-photon counting avalanche photodiode (PDM Series, MPD, PicoQuant) and timed using a single-photon counting module (PicoHarp 300, PicoQuant). n=40-60 cells per time point for each condition were obtained in one experiment.

Confocal FLIM experiments to measure EGFR phosphorylation were performed on a Leica SP8 confocal microscope equipped with a pulsed 470-670 nm white light laser (white light laser Kit WLL2, NKT Photonics) (see: Confocal microscopy) at 514 nm with a pulse frequency of 20 MHz and emission was restricted with an Acousto-Optical Beam Splitter (AOBS) to 525-550nm. MCF7 cells transfected with EGFR-mCitrine, PTB-mCherry and cCbl-BFP were pulsed for 5min with EGF-Alexa647 (200ng/ml) using the CellASIC ONIX Microfluidic Platform (Millipore) followed by a washout. FLIM measurements were performed prior to and after 5min of EGF stimulation, as well as every 5min after EGF washout for a total of 30min. N=3 independent experiments were performed per condition.

For all the confocal FLIM experiments, SymPhoTime software V5.13 (PicoQuant) was used to obtain images after an integration time of 2-4min, collecting app. $\sim$ 3.0–5.0x10^6 photons per image. For each pixel, the single photon arrival times of the TCSPC measurement were used to calculate the complex Fourier coefficients of the first harmonic and were corrected by the Fourier coefficient of a calculated reference (Grecco et al., 2010).

Confocal Microscopy

Confocal images were recorded using an Olympus FluoView FV1000 confocal microscope or a Leica SP8 confocal microscope (Leica Microsystems). The Olympus FluoView FV1000 confocal microscope was equipped with a temperature controlled CO_2 incubation chamber at 37°C and a 60x/1.35 NA Oil UPLSApo objective (Olympus Life Science). Fluorescent fusion proteins with BFP, mTFP and mCitrine were excited using the 405nm Diode-UV laser (FV5-LD05, Hatagaya) and the 458/488nm lines of an

Argon-laser (GLG 3135, Showa Optronics). Cy3.5/Alexa568 were excited with a 561nm DPSS laser (85-YCA-020-230, Melles Griot) and Alexa647 was excited with a 633nm He-Ne laser (05LHP-991, Melles Griot). Detection of fluorescence emission was restricted as following: BFP (425-450nm), mTFP (472-502nm), mCitrine (525-555nm), Cy3.5/Alexa568 (572-600nm), Alexa647 (655-755nm). Scanning was performed in frame-by-frame sequential mode with 3x frame averaging and a pinhole of 2.5 airy units.

The Leica TCS SP8 confocal microscope (Leica Microsystems) was equipped with an environment-controlled chamber (Life Imaging Services) maintained at 37°C, an HC PL APO 63x/1.4NA CS2 oil objective and an HC PL APO 63x/1.2NA motCORR CS2 water objective (Leica Microsystems). mCitrine, mCherry and Alexa647 were excited with a 470–670nm white light laser (white light laser Kit WLL2, NKT Photonics) at 514nm, 561nm and 633nm, respectively. mTFP was excited by the 458nm Argon laser line, cHyPer3 and PF6-AM by the 488nm line, while BFP was excited with a 405nm diode laser. Detection of fluorescence emission was restricted with an Acousto-Optical Beam Splitter (AOBS): BFP (425-448nm), mTFP (470-500nm), mCitrine (525-551nm), mCherry (580-620nm), Alexa647 (655-720nm) and cHyPer3 (495-530nm). Notch filters 458/514 and 488/561/633 were used to suppress laser reflection where applicable. When the oil objective was used, the pinhole was set to 3.14 airy units and 12-bit images of 512x512 pixels were acquired in frame sequential mode with 2x frame averaging. The water objective was used for live cell EGFR-mCitrine trafficking experiments and the pinhole was adjusted (ranging from 3.44 to 2.27 airy units) for each separate channel to maintain optical sectioning fixed to 2.5um.

Imaging EGFR Vesicular Dynamics

Confocal laser scanning microscopy of live MCF7 cells was done on a Leica SP8 confocal microscope (Leica Microsystems) at 37°C using a 63x/1.2NA water objective in DMEM (with 25mM HEPES, without Phenol Red). A temperature-controlled in-house-developed (Masip et al., 2016) flow-through chamber was used to administer a 5min pulsed 200ng/ml EGF-Alexa647 stimulus with the aid of a neMESYS low-pressure syringe pump (Cetoni GmbH). Media were exchanged with a constant flow rate of 3μL/s to avoid cell detachment, while a constant flow with a low rate of 1μL/s was maintained for the rest of the experiment. Sustained 200ng/ml EGF-Alexa647 stimulus was administered in 8-well Lab-Tek dishes by pipetting. Images were acquired for ~120min at 1min time intervals. STMs were calculated as described below. The fraction of liganded EGFR-mCitrine was estimated by the EGF-Alexa647/EGFR-mCitrine ratio normalized to the value at 5min, whereas the ligandless EGFR-mCitrine fraction by 1-[EGF-Alexa647/EGFR-mCitrine]. The fraction of phosphorylated EGFR at the plasma membrane was estimated using the translocation of PTB-mCherry to the plasma membrane localized EGFR-mCitrine. The following quantity was normalized: $(PTB_{PM}/PTB_T - PTB_{endo})/EGFR_{PM}/EGFR_T$, where PTB_{PM} is the PTB-mCherry fluorescence at the plasma membrane, PTB_T is the total PTB-mCherry fluorescence in the cell, $EGFR_{PM}$ – the EGFR-mCitrine fluorescence at the plasma membrane, $EGFR_T$ – the total EGFR-mCitrine fluorescence in the cell and PTB_{endo} – the PTB-mCherry fluorescence on vesicular structures in the cytoplasm. PTB_{endo} was estimated from the cytosol by intensity thresholding (1.5*SD percentile) and removed from the PTB_T as it is already bound to the phosphorylated EGFR-mCitrine on endosomes. Similarly, the STMs of phosphorylation were estimated by $(PTB_{PM}/PTB_T)/(EGFR_{PM}/EGFR_T)$ normalized to the phosphorylated plasma membrane fraction of EGFR as estimated above. N=2-3 independent experiments were performed per condition, yielding 12-14 cells per condition.

Multiple EGF Pulse Experiment

MCF7 cells were transfected with PTPRG or Control siRNA and subsequently with EGFR-mCitrine, PTB-mCherry and cCbl-BFP expression plasmids. For the Rab11^{S25N} experiment, Rab11^{S25N}–mTFP was transfected additionally, without siRNA transfection, and the flow-through chamber protocol was used as in the single-pulse vesicular dynamics experiment. For the siRNA experiments, the cells were transferred to CellASIC ONIX microfluidic switching plate (M04S-03, Millipore) in complete growth media for at least 3h followed by serum starvation for at least 6h. An EGF pulse-washout program consisting of a 5min pulse of EGF-Alexa647 (20ng/ml) followed by continual perfusion with serum-free media for 25min was delivered using the CellASIC ONIX microfluidic device. Confocal imaging at 1min time interval was performed concurrently during 4 successive EGF pulse-washout programs using the Leica TCS SP8. Plasma membrane phosphorylated fraction of EGFR-mCitrine was estimated in the same manner as for the single-pulse vesicular dynamics experiment. N=1-3 independent experiments were performed.

QUANTIFICATION AND STATISTICAL ANALYSIS

Single Cell Segmentation and Quantification

Cells were segmented in CellProfiler (Kamentsky et al., 2011) using the image of the nuclear stain (Hoechst) and EGFR-mTFP. The average background was obtained from a cell free area and substracted from all images. Images were also corrected for bleed through, and the mean values per cell (excluding the nuclear region) from all channels were obtained. To match the images of the FLIM MCP and the high-resolution CCD camera, the masks were affine transformed (OpenCV).

Global Analysis of FLIM Data

The Fourier coefficients obtained from the FLIM datasets were analyzed by global analysis as previously described (Grecco et al., 2010). Briefly, the global fluorescence lifetimes of the donor alone (τ_D) and donor paired with acceptor (τ_{DA}) were calculated from

the intersection of a linear fit through the Fourier coefficients determined at each pixel with the semicircle corresponding to mono-exponential decays. For each pixel, the local fraction of donor molecules that exhibit FRET (α) was calculated from the projection onto the fitted line (Figure S2A).

CA-FLIM Identification of PTPs that Dephosphorylate EGFR

EGFR phosphorylation and the respective phosphorylation fold change (PFC) upon ectopic expression and knockdown of individual PTP_Xs were calculated as $\alpha_{PFC} = \alpha_{PTPx}/\alpha_{ctr}$. The corresponding distributions ($\alpha_{median,\ PTPx}$, $\alpha_{median,\ ctr}$) obtained from single cells were subjected to a two-sided Kolmogorov-Smirnov (KS) test (SciPy). In case of ectopic PTP_X-mCitrine expression, if $p < 0.05$ in > 50% of the experiments (N=4–8), the mean α_R was calculated from all significant experiments, otherwise α_R was set to 1.

Relative Specific PTP_X -mCitrine Activity

The α_{median} of each cell was plotted against the respective PTP_X–mCitrine mean intensity per cell for each time point (Figure S2D). If the distributions of $\alpha_{median,\ PTPx}$ and $\alpha_{median,\ ctr}$ were significantly different (Mann–Whitney U, $p<0.05$), the data was fitted with an exponential function ($\alpha = c + A \cdot e^{-k \cdot PTPx}$). For each time point, the cells of the respective control measurement were included in the fit after removing outliers (± 3x median absolute deviation around the median). The relative specific activity of each PTP_X–mCitrine was determined from the slope of the exponential function at 0 calculated from –k*A, where k is the rate and A is the amplitude. For weak α - PTP_X-mCitrine intensity dependencies, the relative specific activity was determined from the slope of a linear fit.

Spatial-Temporal Maps (STMs)

Cells were masked from the EGFR images using FIJI (https://fiji.sc/), the nuclei were segmented using CellProfiler from the nuclear stain (Hoechst) or cCBL-BFP images. For each pixel within the cell, the distance to the closest plasma membrane and nuclear membrane were calculated to derive a normalized distance $r = r_{PM} / (r_{PM} + r_{NM})$. All pixels were split in 10 intervals according to their normalized distances. For each of the observables (EGFR-mTFP, PTP_X-mCitrine, pY_{1068}-Alexa568, and EGF-Alexa647 fluorescence intensities) or derived quantities (α, pY_{1068}-Alexa568/EGFR-mTFP, EGF-Alexa647/ EGFR-mTFP, PFC), the mean value was calculated for each segment, yielding a radial profile for the individual cells. To calculate the radial distribution of EGFR-mTFP phosphorylation, the mean fluorescence per segment of the pY_{1068}-Alexa568 channel was divided by the corresponding mean EGFR-mTFP fluorescence. With the exception of α and pY_{1068}-Alexa568/EGFR-mTFP images, all profiles were divided by the total cell mean and an average radial profile was calculated. The radial profiles from the distinct time points were then combined to yield the corresponding spatial-temporal maps. Cells in which PTP_X-mCitrine expression levels saturated EGFR dephosphorylation were excluded from the analysis (Figure S3D, explanation below).

The STM of the phosphorylation fold-change (PFC) was calculated by dividing the STM pY_{1068}-Alexa568/EGFR-mTFP of the control by the STM pY_{1068}-Alexa568/EGFR-mTFP for each PTP_X-mCitrine. The profiles from all experiments were averaged and significance was determined using $k\sum_{i=0}^{n-1}\frac{(-\ln(k))^i}{i!}$, where $k = \prod_n p_i$, and p_i denotes the individual p-values from a Student's t-test comparing the pY_{1068}-Alexa568/EGFR-mTFP distributions of the control to that upon the respective PTP_X-mCitrine expression at each point in space and time. To obtain the PFC significance, the mean±SD for each STM of pY_{1068}-Alexa568/EGFR-mTFP was calculated per batch, for both the control case and upon PTP_X perturbation (cDNA or siRNA). Statistical significance analysis between the two cases was carried out for every spatio-temporal point independently, assuming the data sets are log-normally distributed. Logarithms of the two variables will then give normally distributed variables, which were subtracted using Gaussian addition, effectively calculating the PFC of the batch induced by the PTP_X perturbation. For the cDNA case, the control pY_{1068}-Alexa568/EGFR-mTFP was divided over the respective STM upon PTP_X-mCitrine expression, whereas for the siRNA case, the ratio was inverted. Using Gaussian product, we then combined the normally distributed variables of the different batches to produce the combined log-PFC. We perform a t-test on this distribution, propagating the degrees of freedom (number of data points), using one-sided Welch's t-test where we checked how statistically significant is the log-PFC distribution relative to zero. To obtain the plots shown in Figures 3B–3D, we convert back to a combined log-normal PFC, that can be described though its mean±SD. The spatio-temporal points that are not significantly larger than one (using out previous t-test results of the log-PFC) are shown as transparent in Figures 3B–3D.

Determining PTP_X Reactivity towards Phosphorylated EGFR

From the EGFR/PTP reaction cycle, the temporal evolution of phosphorylated ligand-bound EGFR ($EGF{-}EGFR_p$) can be described by:

$$\frac{d[EGF - EGFR_p]}{dt} = k_{EGFR}\left([EGFR]_T - [EGF - EGFR_p]\right) - [EGF - EGFR_p]\left(k_{PTP_X}[PTP_X] + [PTP_e]\right) \quad \text{(Equation 1)}$$

where the forward kinase reaction is assumed to be first order and the backward reaction second order. k_{EGFR} is the rate constant of kinase activity, k_{PTPX} - the PTP_X-mCitrine dephosphorylation rate constant, $[PTP_X]$ the concentration of PTP_X-mCitrine, $[EGFR]_T$ the

total EGFR concentration and $[PTP_e]$ the overall endogenous PTP_e activity. Assuming that the local steady-state is reached on a shorter time scale than exchange of EGFR between denoted spatial segments via trafficking (Hendriks et al., 2003), gives:

$$k_{PTP_X}[PTP_X]+[PTP_e]=k_{EGFR}\frac{[EGFR]_T-[EGF-EGFR_p]^*}{[EGF-EGFR_p]^*}=k_{EGFR}\left(\frac{1}{\left(\frac{[EGF-EGFR_p]^*}{[EGFR]_T}\right)_{PTP_X}}-1\right) \quad \text{(Equation 2)}$$

with $\frac{[EGF-EGFR_p]^*}{[EGFR]_T}$ being the fraction of phosphorylated liganded EGFR. In the absence of ectopic PTP_X-mCitrine expression ($[PTP_X]=0$), the overall endogenous PTP activity is $[PTP_e]=k_{EGFR}\left(\frac{1}{\left(\frac{[EGF-EGFR_p]^*}{[EGFR]_T}\right)_{ctr}}-1\right)$, rendering the reactivity of PTP_X–mCitrine towards specific EGFR-pY_{1068} to be:

$$\frac{k_{PTP_X}[PTP_X]}{k_{EGFR}}=\frac{1}{\left(\frac{[EGF-EGFR_p]^*}{[EGFR]_T}\right)_{PTP_X}}+\frac{1}{\left(\frac{[EGF-EGFR_p]^*}{[EGFR]_T}\right)_{ctr}} \quad \text{(Equation 3)}$$

for each spatial-temporal bin. Additionally,

$$\left(\frac{[EGF-EGFR_p]^*}{[EGFR]_T}\right)_{PTP_X}=\frac{1}{\frac{k_{PTP_X}}{k_{EGFR}}[PTP_X]+\frac{1}{\left(\frac{[EGF-EGFR_p]^*}{[EGFR]_T}\right)_{ctr}}} \quad \text{(Equation 4)}$$

depicts the dependency of the fraction of phosphorylated EGFR on the PTP_X-mCitrine expression level in steady state and was used to determine PTP_X-mCitrine expression levels where EGFR phosphorylation was sensitive to the perturbation (Figure S3D).

Ligandless EGFR Recycling Rates

To determine the recycling dynamics of ligandless EGFR upon 5min pulsed EGF stimulus, we developed a dual-compartment model where EGFR internalization from the plasma membrane to the recycling endosome occurs with rate constant k_{in} and EGFR recycling back to the PM with rate constant k_{rec}. During the initial 5min stimulus, the plasma membrane fraction of ligandless EGFR ($EGFR_{PM}$) relative to the total ligandless concentration ($EGFR_T$) is reduced due to ligand binding. Assuming no further conversion between ligandless and liganded EGFR occurs after removal of EGF, replenishing ligandless EGFR at the plasma membrane takes place in the time span of ~4-35min according to the following dynamics:

$$\frac{d[EGFR_{PM}]}{dt}=k_{rec}\left([EGFR]_T-[EGFR_{PM}]\right)-k_{in}[EGFR_{PM}] \quad \text{(Equation 5)}$$

yielding a closed-form solution

$$\left(\frac{[EGFR_{PM}]}{[EGFR]_T}\right)(t)=\frac{k_{rec}}{k_{rec}+k_{in}}-\left(\frac{k_{rec}}{k_{rec}+k_{in}}-\left(\frac{[EGFR_{PM}]}{[EGFR]_T}\right)(t_0)\right)e^{-(k_{rec}+k_{in})(t-t_0)} \quad \text{(Equation 6)}$$

Here, $\left(\frac{[EGFR_{PM}]}{[EGFR]_T}\right)(t)$ represents the fraction of EGFR at the plasma membrane for a particular time t, and $\left(\frac{[EGFR_{PM}]}{[EGFR]_T}\right)(t_0)$ - the plasma membrane fraction at $t_0 \approx 5$min. This model was used to infer the trafficking rates from the live cell data, where the first three (out of ten) spatial bins defined the plasma membrane (Figure 1G, bottom). Given that in steady state $\left(\frac{[EGFR_{PM}]}{[EGFR]_T}\right)^*=\frac{k_{rec}}{k_{rec}+k_{in}}$, renders

$$k_{in}=k_{rec}\frac{1-\left(\frac{[EGFR_{PM}]}{[EGFR]_T}\right)^*}{\left(\frac{[EGFR_{PM}]}{[EGFR]_T}\right)^*} \quad \text{(Equation 7)}$$

Thus, the steady state plasma membrane fraction of ligandless EGFR estimated from the k_{in} vs k_{rec} correlation scatter plot (Figure S1K) was 0.43 with 95% confidence bounds (0.37, 0.49). The estimated average quantities (with 95% confidence bounds) were: k_{in} = 0.31min^{-1} (0.12, 0.50), k_{rec}=0.23min^{-1} (0.08, 0.38), and the recycling half-life $t_{1/2}=\frac{ln2}{k_{rec}+k_{in}}$ = 4.32min (1.02, 7.62).

Live Cell Dose Response Imaging and Quantification

Confocal laser scanning microscopy on live MCF7 cells was performed on a Leica SP8 confocal microscope (Leica Microsystems) using a 63x/1.4NA oil objective. The samples were maintained at 37°C in DMEM (with 25mM HEPES, without Phenol Red). Cells were stimulated every ~1.5min with increasing dose of EGF-Alexa647, ranging from 2.5ng/ml to 600ng/ml (0.34nM to 81.29nM; doses

were roughly doubled: D={2.5, 7.2, 16.4, 34.75, 71.6, 145.6, 294.4, 593.4ng/ml}). For NOX inhibition, cells were incubated with 10μM DPI for 30min prior to stimulation (N=4-12 independent experiments were performed per condition). The fluorescence of expressed TagBFP was used to identify the cytoplasmic region of the cell using Otsu's thresholding method (Otsu, 1979) (scikit-image, scikit-image.org). The plasma membrane region of a cell in each time point was calculated by subtracting the cytoplasmic region from the cellular image mask.

PTB–mCherry translocation to (pY_{1086}, pY_{1148}) PM-bound EGFR-mTFP(mCitrine) for a given EGF-Alexa647 dose $d \in D$ was quantified as:

$$PTB - EGFR(d) = \frac{[PTB_{PM}]/[PTB]_T}{[EGFR_{PM}]/[EGFR]_T}(d) \qquad \text{(Equation 8)}$$

where $[PTB_{PM}]$ is the PTB-mCherry translocated to the plasma membrane, whereas $[PTB]_T$ is the total PTB-mCherry in the cell. The fraction of phosphorylated receptor was then calculated by normalizing this value between the initial (unstimulated) and maximal value of the series

$$[pEGFR](d) = \frac{PTB - EGFR(k) - PTB - EGFR(0)}{\max_i PTB - EGFR(i) - PTB - EGFR(0)} \qquad \text{(Equation 9)}$$

where pEGFR refers to the fraction of phosphorylated EGFR. Similarly, the amount of liganded receptor for dose d was calculated from the ratio of integrated EGF-Alexa647 and EGFR-mTFP(mCitrine) fluorescence at the plasma membrane:

$$[EGF - EGFR](d) = \frac{[EGF_{PM}]}{[EGFR_{PM}]}(d) \qquad \text{(Equation 10)}$$

The fraction of liganded receptor (lEGFR) was calculated as:

$$lEGFR(d) = \frac{[EGF - EGFR](k) - [EGF - EGFR](0)}{\max_i [EGF - EGFR](i) - [EGF - EGFR](0)} \qquad \text{(Equation 11)}$$

To estimate the relation between the fraction of ligand-bound receptor and the actual administered EGF dose (Figure 1D), the following ligand-binding kinetics model was used:

$$[EGFR] + [EGF] \underset{k_r}{\overset{k_f}{\rightleftharpoons}} [EGF - EGFR] \qquad \text{(Equation 12)}$$

with $K_D = \frac{k_r}{k_f}$ being the dissociation constant. Assuming that at low EGF doses, the ligand will be depleted from the solution due to binding to EGFR (Lauffenburger and Linderman, 1996), the fraction of ligand bound receptor in steady state gives the following closed-form solution:

$$\frac{[EGF - EGFR]}{[EGFR]_T} = \frac{\frac{n}{N_A}[EGFR]_T + [EGF]_T + K_D - \sqrt{\left(\frac{n}{N_A}[EGFR]_T + [EGF]_T + K_D\right)^2 - 4\frac{n}{N_A}[EGFR]_T[EGF]_T}}{2\frac{n}{N_A}[EGFR]_T} \qquad \text{(Equation 13)}$$

where $[EGFR]_T$ = $[EGFR]+[EGF-EGFR]$ - the total EGFR concentration on the plasma membrane and $[EGF]_T = [EGF] + \frac{n}{N_A}[EGF - EGFR]$ - the total input EGF dose, n is the number of cells, and N_A is Avogadro's number needed for converting the number of ligand-bound receptors into moles. This function was used to fit the experimental data (Figures S1B and S1C) thereby mapping the input dose to a fraction of ligand-bound receptor. K_D was obtained to be 762pM (427, 1097) with 95% confidence bounds.

Area under the curve (AUC) of the dose-response profile of each cell was used as an integrated measure of the response function. The distributions of AUC values between two datasets were compared using two-sample Student's t-test.

Modeling EGFR Phosphorylation Dynamics

Using the conservation of mass balances:

$$[EGF - EGFR]_T = 2[EGF - EGFR] + 2[EGF - EGFRp]$$

$$[EGFR]_T = [EGF - EGFR]_T + [EGFRp] + [EGFR], \qquad \text{(Equation 14)}$$

$$[PTP]_T = [PTPa] + [PTPi]$$

where $[EGF{-}EGFR]_T$ is the total amount of ligand-bound receptor, $[EGF{-}EGFR]$ - non-phosphorylated ligand-bound dimeric EGFR, $[EGF{-}EGFRp]$ –phosphorylated ligand-bound dimeric EGFR, $[EGFR]$ - ligandless non-phosphorylated EGFR, $[EGFRp]$ - ligandless phosphorylated EGFR and $[PTP]_T$ the total amount of ectopically expressed active (PTP_a) and inactive (PTP_i) PTP. The reaction networks from Figure S4A can be therefore described by the generalized model (Equation 15):

$$\frac{d[EGFRp]}{dt} = \left([EGFR]_T - [EGF-EGFR]_T - [EGFRp]\right)\left(\alpha_1\left([EGFR]_T - [EGF-EGFR]_T - [EGFRp]\right) + \alpha_2[EGFRp] + \alpha_3[EGF-EGFRp]\right) - [EGFRp](\gamma[PTPa] + [PTPe])$$

$$\frac{d[EGF-EGFRp]}{dt} = k_5\left([EGF-EGFR]_T/2 - [EGF-EGFRp]\right) - [EGF-EGFRp](\gamma[PTPa] + [PTPe]) \quad \text{(Equation 15)}$$

$$\frac{d[PTPa]}{dt} = k_1\left([PTP]_T - [PTPa]\right) - k_2[PTPa] - k_3[PTPa](2[EGF-EGFRp] + [EGFRp]) + k_4\left([PTP]_T - [PTPa]\right) \times (2[EGF-EGFRp] + [EGFRp])$$

with PTPe contribution given as:

$$[PTPe] = c + \frac{1}{a + b(2[EGF-EGFRp] + [EGFRp])} \quad \text{(Equation 16)}$$

To describe the aggregated effect of endogenous PTP activity on EGFR phosphorylation, the quantities describing ectopic PTP_X expression are set to 0, and a,b and c are arbitrary parameters that approximate the aggregated activity of multiple endogenously expressed PTPs. This overall activity was modelled as a combination of double-negative and negative feedback topology as well as negative regulation motifs. In case of ectopic PTP_X-mCitrine expression on the other hand ($\gamma > 0$), dephosphorylation of EGFR by PTP_X-mCitrine will dominate over PTP_e, therefore allowing to set $[PTP_e]$ to zero. Additionally, the following parameter restrictions were imposed: double-negative feedback ($k_3 > 0$, $k_4 = 0$), negative feedback ($k_3 = 0$, $k_4 > 0$) or negative regulation ($k_3 = 0$, $k_4 = 0$).

To determine which of the three EGFR-PTP network topologies (Figure S4A) best represents the experimental EGF dose - EGFR phosphorylation responses upon ectopic PTP_X-mCitrine expression, the model and data were transformed by expressing the dependency of the fraction of phosphorylated EGFR ($[pEGFR] = (2[EGF-EGFRp] + [EGFRp])/[EGFR]_T$) to the fraction of liganded EGFR-mTFP. The models were then fitted to the data, and the parameters were estimated using an adaptive Metropolis-Hastings algorithm, a variant of the Monte Carlo Markov Chain (MCMC) method for sampling from the posterior joint probability distribution of the parameters (Chib and Greenberg, 1995). Akaike information criterion was used for model discrimination (Hipel, 1981). The parameter values used to fit all EGF-dose EGFR-response curves in Figures 4A–4C, and the corresponding Akaike information criterion values are shown in Table S1. The analysis was performed with an in-house developed code in MATLAB (The MathWorks, Inc).

To describe the dynamics of the effective EGFR-PTP network at the PM (Figures 5B and 5D), the double-negative feedback model (Equation 15) was extended with:

$$\frac{d[PTPN2_a]}{dt} = \varepsilon * \left(k_4 * [EGFRp] * \left([PTPN2]_T - [PTPN2_a]\right) - k_2 * [PTPN2_a]\right) \quad \text{(Equation 17)}$$

The dephosphorylation of [EGFRp] by PTPN2 was described by an additional term in Equations 15–1: $[\text{EGFRp}] * \gamma_1 * [\text{PTPN2}_a]$. The EGFR-PTPN2 negative feedback is on a time scale (ϵ) approximately two orders of magnitude slower than the phosphorylation-dephosphorylation reaction, as estimated from the ~4min recycling time (Figure S1K). The bifurcation analysis of this network was performed using the Bifurcation analysis software XPPAUT (www.math.pitt.edu/~bard/xpp/xpp.html) and interpolated in MATLAB to generate 3D diagrams shown in Figures 5C–5D.

DATA AND SOFTWARE AVAILABILITY

All data and software used in this manuscript are available upon request, for contact information see section ‘Contact for Reagent and Resource Sharing’.

Cell Systems, Volume 7

Supplemental Information

Interdependence between EGFR and Phosphatases Spatially Established by Vesicular Dynamics Generates a Growth Factor Sensing and Responding Network

Angel Stanoev, Amit Mhamane, Klaus C. Schuermann, Hernán E. Grecco, Wayne Stallaert, Martin Baumdick, Yannick Brüggemann, Maitreyi S. Joshi, Pedro Roda-Navarro, Sven Fengler, Rabea Stockert, Lisaweta Roßmannek, Jutta Luig, Aneta Koseska, and Philippe I.H. Bastiaens

Supplemental Information

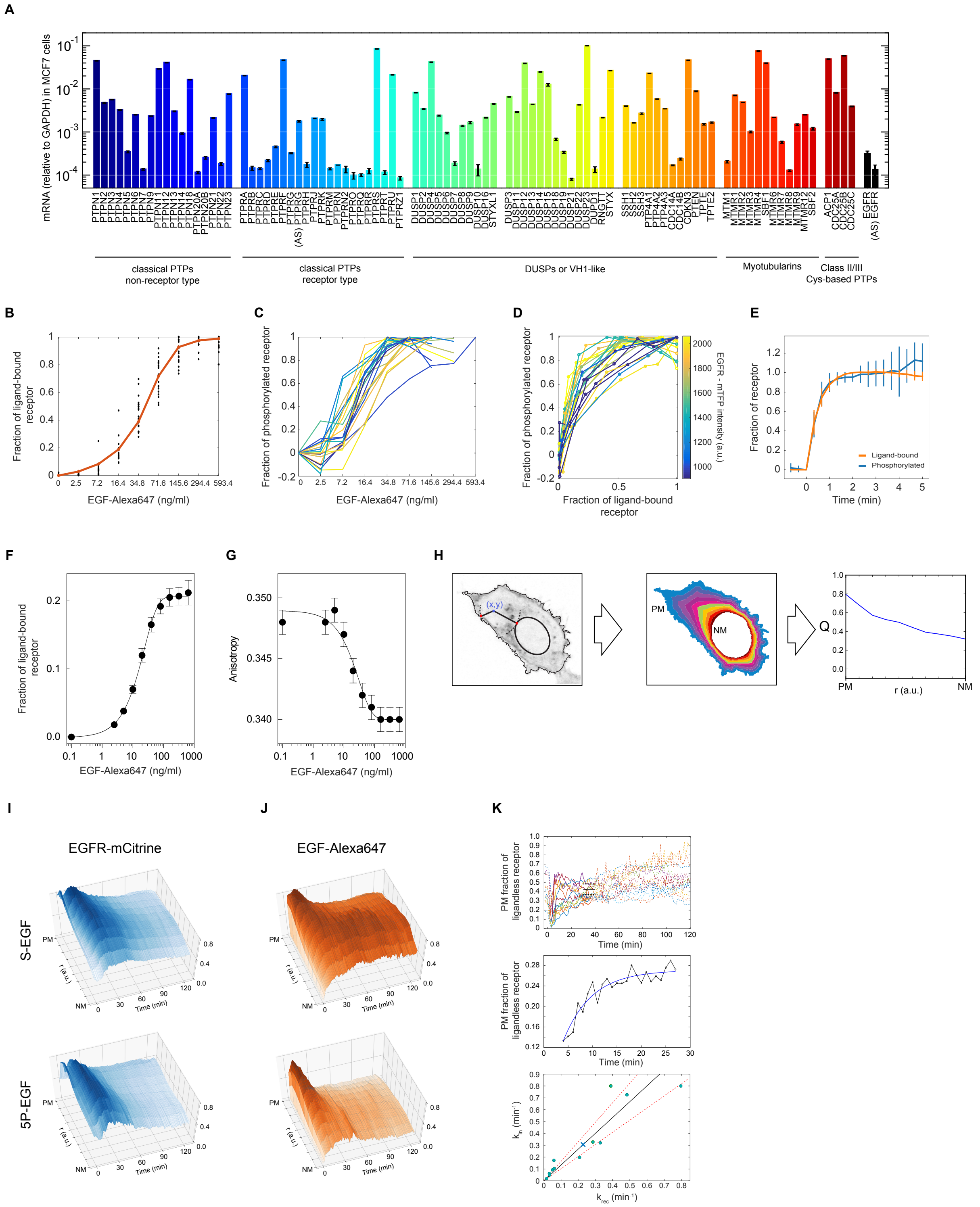

Figure S1. EGFR phosphorylation and trafficking dynamics, related to Figure 1. (A) PTP_X and EGFR mRNA expression relative to GAPDH mRNA in MCF7 cells (means±SD) measured by microarray analysis. AS: anti-sense. **(B)** Estimating fraction of EGFR-mTFP bound to EGF-Alexa647 in live cells: EGF-Alexa647/EGFR-mTFP quantified in single cells (data points) upon increasing EGF-Alexa647 doses was fitted (line) with a receptor binding

kinetics model (STAR Methods). **(C)** Single cell profiles of the fraction of phosphorylated EGFR-mTFP, quantified by PTB-mCherry translocation to the plasma membrane ($\frac{[PTB-mCherry_{PM}]/[PTB-mCherry]_T}{[EGFR-mTFP_{PM}]/[EGFR-mTFP]_T}$, STAR Methods) for increasing EGF-Alexa647 doses. **(D)** Single cell EGFR-mTFP phosphorylation response versus fraction of ligand bound receptors (mean±SD shown in Figure 1D) derived from (**B**) and (**C**). The estimated fractions of phosphorylated vs. liganded EGFR-mTFP are plotted and color-coded by the average EGFR-mTFP fluorescence intensity per cell. **(E)** Quantification of PTB-mCherry translocation kinetics to plasma membrane localized EGFR-mTFP. MCF7 cells were stimulated with a saturating EGF-Alexa647 dose (320ng/ml) and successive images were acquired every 20s (n=10, means±SD). Translocated plasma membrane fraction of PTB-mCherry ($\frac{[PTB-mCherry_{PM}]/[PTB-mCherry]_T}{[EGFR-mTFP_{PM}]/[EGFR-mmTFP]_T}$) converged to a steady state level in ~1.5min, which was within the time frame of successive EGF-Alexa647 dose administration (Figure 1C-D). **(F)** Estimation of the fraction of EGFR-QG-mCitrine bound to EGF-Alexa647 in live cells (n=30, N=3, means±SEM) from fluorescence anisotropy microscopy is equivalent to the corresponding estimation from confocal microscopy with EGFR-mTFP (Figure S1B). **(G)** Dependency of the EGFR-QG-mCitrine dimerization state on increasing EGF-Alexa647 doses determined by fluorescence anisotropy microscopy (n=30, N=3, means±SEM). **(H)** Dimensionality reduction from Cartesian (x, y) to normalized radial (r) distribution of quantity (Q) between the plasma (PM) and the nuclear (NM) membrane. **(I)** Average spatial-temporal maps (STMs) of EGFR-mCitrine intensity obtained from live cells stimulated with 200ng/ml S-EGF (n=16, N=3; top) and 5P-EGF (n=14, N=2; bottom). **(J)** Corresponding STMs of EGF-Alexa647 fluorescence. **(K)** Quantification of recycling dynamics of ligandless EGFR-mCitrine upon 200ng/ml 5P-EGF. Top: plasma membrane fraction of ligandless EGFR-mCitrine in single cells. Model-based estimation of the steady state level (95% confidence bounds; see STAR Methods) is shown with black line (inside red dashed lines). Middle: compartment-model-based fitting on 4-35min time interval for the cells shown in the bottom panel of Figure 1G (estimated rates: k_{in}=0.31min^{-1} (0.12, 0.50) 95% confidence bounds, k_{rec}=0.23min^{-1} (0.08, 0.38), STAR Methods). Bottom: Linear dependency between (k_{in}, k_{rec}) reflects that similar steady state plasma membrane fraction of ligandless EGFR are maintained in different cells by recycling (x: average (k_{in}, k_{rec}); STAR Methods). Linear fit (black line) with 95% confidence interval half-width (dashed red lines) is shown.

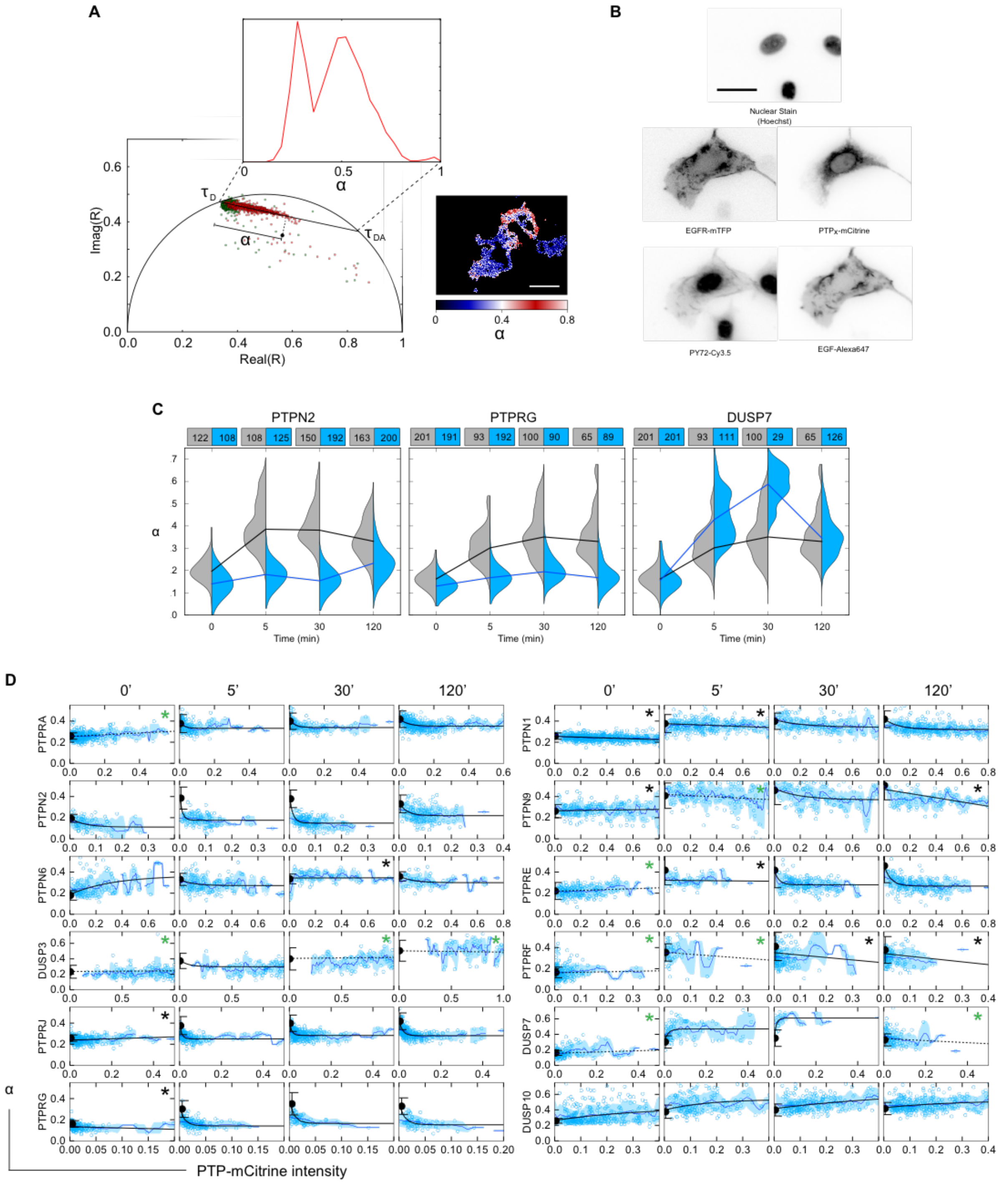

Figure S2. CA-FLIM and related quantities, Related to Figure 2. (A) Linear fit of the fluorescence emission Fourier coefficients (R) in the complex plane yielding the global EGFR-mTFP lifetimes in presence (τ_{DA}) and absence (τ_D) of FRET. Fraction of phosphorylated EGFR-mTFP bound by PY72-Cy3.5 (α) in each pixel was calculated from the projection onto the τ_D-τ_{DA} line segment. An exemplary α-histogram (inset) and a spatially resolved α-map (right) are shown. Scale bar: 20µm. **(B)** Representative images obtained in CA-FLIM screen: Hoechst (nuclear stain), EGFR-mTFP, PTP_X-mCitrine, PY72-Cy3.5 and EGF-Alexa647 fluorescence. Scale bar: 10µm. **(C)** Exemplary temporal EGFR-mTFP

phosphorylation profiles (grey, control) upon ectopic PTP_X-mCitrine expression (PTP_X=PTPN2, PTPRG, DUSP7; blue). The violin plots show the α distributions from single cells stimulated with 200ng/ml 5P-EGF (number of cells denoted on top of the plots, medians at different time points are connected by a black line). **(D)** α vs. PTP_X-mCitrine single cell fluorescence scatter plots. Black circle: mean α_{ctr}±SD; black lines: exponential fits (* - linear fit for weak dependence, green asterisk: distributions of α_{ctr} and α_{PTP} did not significantly differ); blue lines with error bounds: moving averages with standard deviations. The slope of the exponential (or linear) fit at the intercept is defined as the relative specific PTP_X-mCitrine activity in Figure 2C (middle).

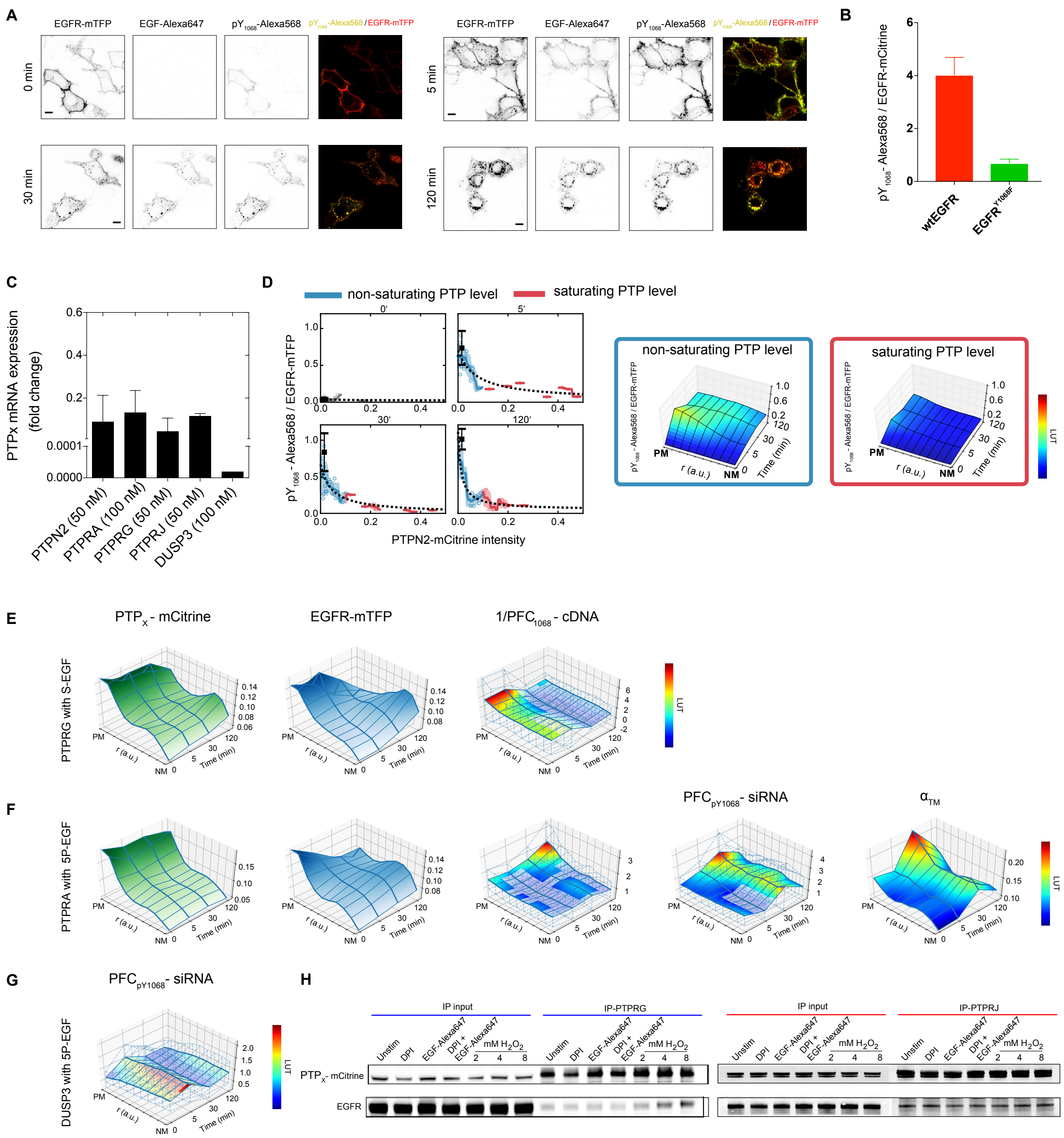

Figure S3. PTPs shape EGFR phosphorylation response, Related to Figure 3. (A) Representative images of EGFR-mTFP, EGF-Alexa647, anti-pY_{1068}-Alexa568 fluorescence and overlay of pY_{1068} (yellow) and EGFR-mTFP (red) prior to and after 5, 30 and 120min of 5P-EGF stimulation (200ng/ml) of MCF7 cells. Scale bar: 50μm. **(B)** Binding of pY_{1068}-Alexa568 antibody to pY_{1068} on EGFR-mCitrine and $EGFR^{Y1068F}$-mCitrine reflects its specificity for the corresponding tyrosine phosphorylation site (mean±SD, n~100, N=1). **(C)** mRNA expression fold change of PTPN2, PTPRG, PTPRJ, PTPRA and DUSP3 in MCF7 cells after 24h transfection with 50nM or 100nM respective siRNA. The values are relative to the corresponding mRNA levels of cells treated with 50nM non-targeting siRNA for 24hr (means±SEM, N=2, and for DUSP3 N=1). **(D)** Left: example of pY_{1068}-Alexa568/EGFR-

mTFP vs. PTPN2-mCitrine fluorescence intensity scatter plots used to determine the PTPN2-mCitrine fluorescence intensity threshold at which saturation of EGFR dephosphorylation occurs. Blue/red circles represent single cells below and above the PTPN2-mCitrine fluorescence intensity threshold, respectively; solid lines and shaded bounds: corresponding moving averages and standard deviation. The data was fitted with a function depicting the dependency of pY_{1068}/EGFR-mTFP on PTP_X-mCitrine intensity (steady state EGFRp assumption, STAR Methods). Right: spatial temporal maps (STMs) of pY_{1068}/EGFR-mTFP averaged from cells below (blue box) and above (red box) PTPN2-mCitrine fluorescence intensity threshold. **(E)** Effect of PTPRG-mCitrine expression (left) on STMs of EGFR-mTFP fluorescence (middle) and phosphorylation fold-change (1/PFC_{pY1068}-cDNA, right) reflecting the relative PTPRG-mCitrine reactivity towards pY_{1068} for cells stimulated with 200ng/ml S-EGF (n~30). **(F)** Columns 1-3: effect of PTPRA-mCitrine expression (Column 1) on STMs of EGFR-mTFP localization (Column 2) and phosphorylation fold-change (1/PFC_{pY1068}-cDNA, Column 3), which reflects the relative PTPRA-mCitrine reactivity towards pY_{1068} (n~60, N=3). Column 4: effect of siRNA-mediated PTPRA knock-down on EGFR-mTFP phosphorylation fold change (PFC_{pY1068}-siRNA, n~45, N=3). Column 5: STM of fraction of EGFR-mTFP interacting with PTPRA ^{C442S}-mCitrine trapping mutant (α_{TM}, n=15-30, N=2). **(G)** Effect of siRNA-mediated DUSP3 knock-down on EGFR-mTFP phosphorylation fold change (PFC_{pY1068}-siRNA, n~40, N=3). In (F-G), cells were stimulated with 200ng/ml 5P-EGF; transparent areas in (E-G): non-significant PFCs, p >0.05. LUT: look-up table. **(H)** Identifying and characterizing the interaction between EGFR and PTPRG/J-mCitrine by co-immunoprecipitation. Co-immunoprecipitated EGFR (second row) following PTPRG- (left) or PTPRJ-mCitrine (right) pull down from MCF7 cells prior to and after treatment with DPI (20min, 10μM), EGF-Alexa647 (10 min, 200ng/ml), DPI pretreatment (20min, 10μM) followed by EGF-Alexa647 (10 min, 200ng/ml) and H_2O_2 (10 min; 2, 4 or 8 mM) by western blotting. IP input: total expressed protein, IP-PTPRG/J: PTPRG/J-mCitrine immunoprecipitated by anti-GFP antibody.

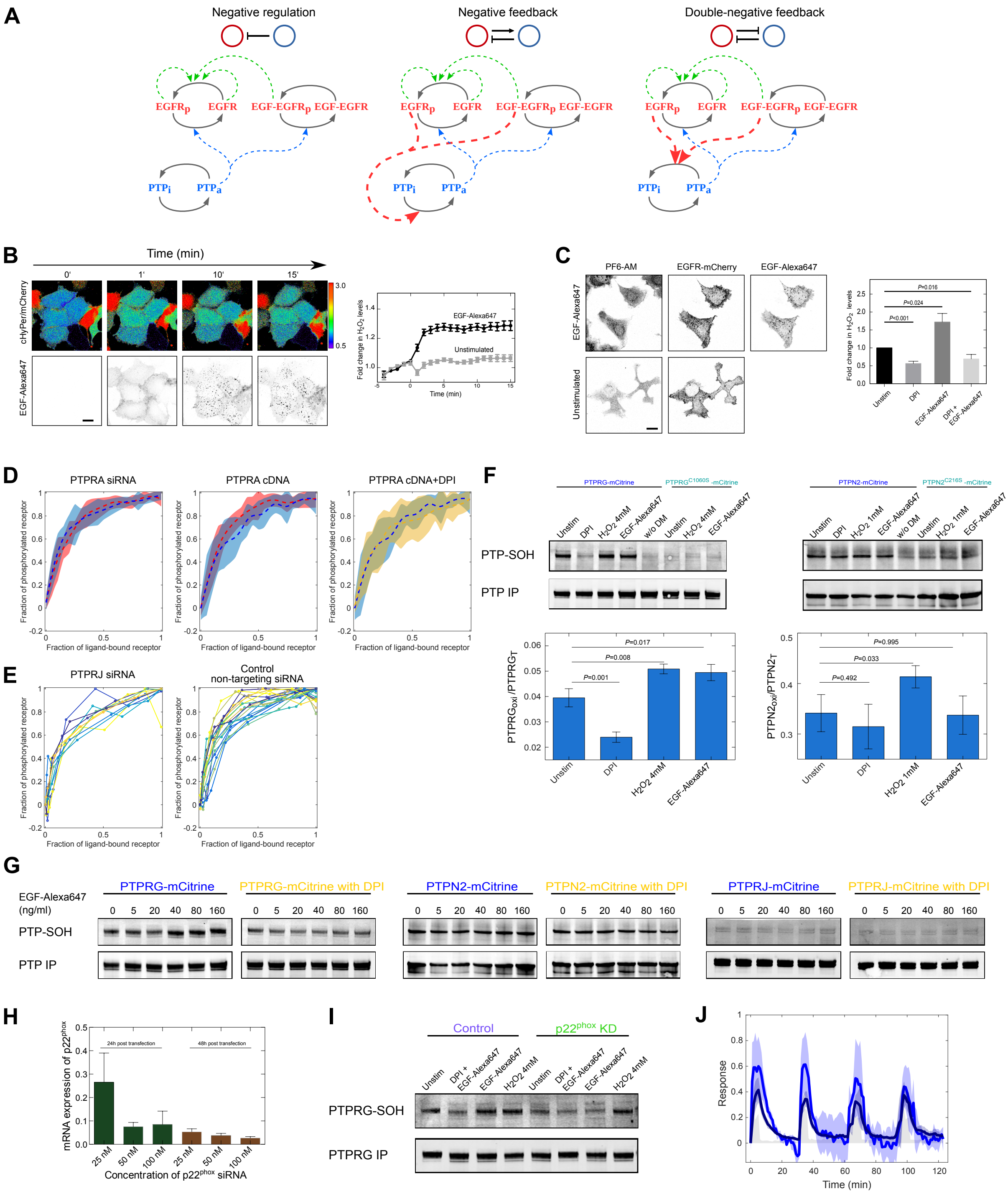

Figure S4. Regulation of EGFR responsiveness by PTPs, Related to Figure 4 and 5. (A) Possible EGFR-PTP network motifs. Solid arrows: molecular state transitions (p: phosphorylation, i: inactive, a: active), dashed arrows: causal links. Left: negative regulation, middle: negative feedback, right: double negative feedback. Top row: corresponding network motifs. **(B)** Left: Representative pseudo-coloured image series of cHyPer3/mCherry fluorescence ratio (upper row), normalized to that at 0min in each MCF7 cell. Images were acquired every minute for 15min after 320ng/ml EGF-Alexa647 stimulation (lower row). Scale bar: 20µm. Right: Corresponding quantification of relative H_2O_2 levels

(cHyPer3/mCherry fluorescence ratio ± SEM) upon EGF-Alexa647 (black line, n=11 cells) or vehicle (unstimulated) administration (grey line, n=9 cells). **(C)** Left: fluorescence images of the H_2O_2-sensitive probe PF6-AM (left), EGFR-mCherry (middle) and EGF-Alexa647 (right) in MCF7 cells with (upper row) and without (lower row) 320ng/ml EGF-Alexa647 stimulation. Scale bar: 20µm. Right: corresponding quantification of H_2O_2 fold-change (mean PF6-AM fluorescence ± SEM from 10 fields of view) upon administration of DPI (10 min, 10µM), EGF-Alexa647 (10 min, 320 ng/ml) or a combination of both. **(D)** Dose-response of EGFR-mTFP phosphorylation (control, red) is not affected upon siRNA-mediated PTPRA knock-down (blue, p=0.823, n=14, N=7, left) or PTPRA-mCitrine co-expression (means±SD, blue, p=0.225, n=34, N=16, middle). NOX-inhibition by DPI (10µM, 30min pre-incubation) has no effect (yellow, p=0.937, n=38, N=16, right) on the dose-response of EGFR phosphorylation upon ectopic PTPRA-mCitrine expression (blue, same as in middle plot). **(E)** Representative dose response curves of EGFR-mTFP phosphorylation in single cells upon siRNA-mediated PTPRJ knock-down (left) and corresponding control with non-targeting siRNA (right). The corresponding means±SD are shown in Figure 4A, bottom. **(F)** Quantification of cysteine oxidation (PTP-SOH) in PTPRG-mCitrine (left) or PTPN2-mCitrine (right) to cysteine sulfenic acid by dimedone conjugation, detected by anti-sulfenic acid modified cysteine antibody. Left: detection of oxidized cysteines in immunoprecipitated PTPRG-mCitrine from MCF7 cells treated for 10 min with DPI (10µM), EGF-Alexa647 (80ng/ml) or 4mM H_2O_2 by western blotting. The weak PTP-SOH bands in the corresponding lanes of the $PTPRG^{C1060S}$-mCitrine mutant show that it is the catalytic cysteine that is oxidized. Right: Oxidation of cysteines in PTPN2-mCitrine and $PTPN2^{C216S}$-mCitrine detected using same conditions as above (means±SEM**,** with the exception of the H_2O_2 dose). **(G)** Cysteine oxidation of PTPRG-mCitrine (left), PTPN2-mCitrine (middle) and PTPRJ-mCitrine (right) upon 10min stimulation with increasing EGF-Alexa647 doses (0-160ng/ml) with and without pretreatment with DPI (20 min, 10µM) (representative WBs). **(H)** mRNA expression of $p22^{phox}$ determined by RT-PCR after 24h or 48h of siRNA transfection with different concentrations (means±SEM**,** N=2). **(I)** Representative WB of cysteine oxidation in PTPRG-mCitrine upon non-targeting (control) or $p22^{phox}$ siRNA transfection. Quantification given in Figure 4E. **(J)** Temporal traces of the fraction of ligand bound (EGF-Alexa647/EGFR-mCitrine, dark) and phosphorylated EGFR estimated by PTB-mCherry translocation to the PM (PTB-mCherry/EGFR-mCitrine, light) in live MCF7 cell following siRNA mediated knock-down of PTPRG (means±SD**,** n=4, N=2). Data was acquired at 1min-intervals following 20ng/ml 5P-EGF every 30min.
